# *Cacaoporus*, a new Boletaceae genus, with two new species from Thailand

**DOI:** 10.3897/mycokeys.54.35018

**Published:** 2019-06-10

**Authors:** Santhiti Vadthanarat, Saisamorn Lumyong, Olivier Raspé

**Affiliations:** 1 Department of Biology, Faculty of Science, Chiang Mai University, Chiang Mai, 50200, Thailand Department of Biology, Faculty of Science, Chiang Mai University Chiang Mai Thailand; 2 PhD’s Degree Program in Biodiversity and Ethnobiology, Department of Biology, Faculty of Science, Chiang Mai University, Chiang Mai, 50200, Thailand Botanic Garden Meise Meise Belgium; 3 Center of Excellence in Microbial Diversity and Sustainable Utilization, Faculty of Science, Chiang Mai University, Chiang Mai, 50200, Thailand Department of Biology, Faculty of Science, Chiang Mai University Chiang Mai Thailand; 4 Meise Botanic Garden, Nieuwelaan 38, 1860 Meise, Belgium Botanic Garden Meise Meise Belgium; 5 Fédération Wallonie-Bruxelles, Service général de l’Enseignement universitaire et de la Recherche scientifique, Rue A. Lavallée 1, 1080 Bruxelles, Belgium Department of Biology, Faculty of Science, Chiang Mai University Chiang Mai Thailand; 6 Academy of Science, The Royal Society of Thailand, Bangkok, 10300, Thailand Botanic Garden Meise Meise Belgium

**Keywords:** 3 new taxa, *atp*6, Boletales, *cox*3, Fungal Diversity, multigene phylogeny, *
Neoboletus
*, *Pulveroboletus* group, Taxonomy

## Abstract

We introduce a new genus, *Cacaoporus*, characterised by chocolate brown to dark brown basidiomata and hymenophore, tubes not separable from the pileus context, white to off-white basal mycelium, reddening when bruised, amygdaliform to ovoid spores and dark brown spore deposit. Phylogenetic analyses of a four-gene dataset (*atp*6, *tef*1, *rpb*2 and *cox*3) with a wide selection of Boletaceae showed that the new genus is monophyletic and sister to the genera *Cupreoboletus* and *Cyanoboletus* in the *Pulveroboletus* group. Two new species in the genus, *C.pallidicarneus* and *C.tenebrosus* are described from northern Thailand. Full descriptions and illustrations of the new genus and species are presented. The phylogeny also confirmed the reciprocal monophyly of *Neoboletus* and *Sutorius*, which further support the separation of these two genera.

## Introduction

In the last decade or so, since molecular techniques and phylogenetic analyses have been used in taxonomy and systematics of the Boletaceae, many new species and genera have been described worldwide (e.g. Halling et al. 2012, 2016; Zeng et al. 2012; Arora and Frank 2014; Gelardi et al. 2014, 2015; Li et al. 2014, Zhao et al. 2014b, Zeng et al. 2014; Wu et al. 2015, 2016; Zhu et al. 2015). In Thailand, although the Boletaceae have been studied for a long time, only a few new Boletaceae species and a new genus have recently been described (Desjardin et al. 2009; Neves et al. 2012; Halling et al. 2014; Raspé et al. 2016; Vadthanarat et al. 2018). At the same time, many new species and genera have been described from southern and south-western China, an area with a climate and forests similar to Thailand (e.g. Li et al. 2011; Wu et al. 2015, 2016; Zhu et al. 2015). Similarly, a high number of new species and possibly new genera are expected to occur in Thailand (Hyde et al. 2018)

During our survey on the diversity of boletes in Thailand, several collections of brown to chocolate to dark brown boletes were obtained. Some collections bearing resemblance to *Sutorius* Halling, Nuhn & N.A. Fechner species, which typically have brown or reddish to purplish-brown basidiomata with reddish to purplish-brown hymenophore, reddish-brown spore deposit and narrowly ellipsoid to ellipsoid basidiospores (Halling et al. 2012). However, our chocolate brown bolete collections also showed differences, in particular in having a darker hymenophore, as well as in some microscopic characters like spore shape. We therefore performed a family-wide phylogeny, which showed that those brown to chocolate to dark brown boletes belong in a generic lineage, different from *Sutorius*. Consequently, we introduce the new Boletaceae genus *Cacaoporus* and describe two new species, *C.pallidicarneus* and *C.tenebrosus*, with full descriptions and illustrations.

## Materials and method

### Specimens collecting

Fresh basidiomata were collected in Chiang Mai Province, northern Thailand during the rainy season in 2013 to 2018. The specimens were photographed *in situ*, wrapped in aluminium foil and taken to the laboratory. After description of macroscopic characters, the specimens were dried in an electric drier at 45–50 °C. Examined specimens were deposited in the herbaria CMUB, MFLU, BKF and BR (listed in Index Herbariorum; Thiers, continuously updated).

### Morphological studies

Macroscopic descriptions were made, based on detailed field notes and photos of fresh basidiomata. Colour codes were taken from Kornerup and Wanscher (1978). Macrochemical reactions (colour reactions) of pileus, pileus context, stipe, stipe context and hymenophore were determined using 10% aqueous potassium hydroxide (KOH) and 28–30% ammonium hydroxide (NH_4_OH). Microscopic structures were observed from dried specimens, using 5% KOH, NH_4_OH, Melzer’s reagent or stained with 1% ammoniacal Congo red. A minimum of 50 basidiospores, 20 basidia and 20 cystidia were randomly measured at 1000× with a calibrated ocular micrometer using an Olympus CX51 compound microscope. The notation ‘[m/n/p]’ represents the number of basidiospores “m” measured from “n” basidiomata of “p” collections. Dimensions of microscopic structures are presented in the following format: (a–)b–c–d(–e), in which “c” represents the average, “b” the 5^th^ percentile, “d” the 95^th^ percentile, “a” the minimum and “e” the maximum. *Q*, the length/width ratio, is presented in the same format. A section of the pileus surface was radially and perpendicularly cut to the surface at a point halfway between the centre and margin of the pileus. Sections of stipitipellis were taken from halfway up the stipe and longitudinally cut, perpendicularly to the surface (Hosen et al. 2013; Li et al. 2011). All microscopic features were drawn by free hand using an Olympus Camera Lucida model U−DA fitted to the microscope cited above. For scanning electron microscopy (SEM), a spore print was mounted on to an SEM stub with double-sided tape. The samples were coated with gold, then examined and photographed with a JEOL JSM–5910 LV SEM.

### DNA isolation, PCR amplification and DNA sequencing

Genomic DNA was extracted from fresh tissue preserved in CTAB or about 10–15 mg of dried tissue using a CTAB isolation procedure adapted from Doyle and Doyle (1990). Portions of the genes *atp*6, *tef*1, *rpb*2 and *cox*3 were amplified by polymerase chain reaction (PCR) and sequenced by Sanger sequencing. The primer pairs ATP6-1M40F/ATP6-2M (Raspé et al. 2016), EF1-983F/EF1-2218R (Rehner and Buckley 2005) and bRPB2-6F/bRPB2-7.1R (Matheny 2005) were used to amplify *atp*6, *tef*1 and *rpb*2, respectively. Part of the mitochondrial gene *cox*3 was amplified with the newly designed primers COX3M1-F (5’-ATYGGAGCWGTAATGTWYATGC-3’) and COX3M1-R (5’-CCWACTAWTACRTGRATWCCATG-3’), using the following PCR programme: 2 min 30 s at 95 °C; 35 cycles of 25 s at 95 °C, 30 s at 48 °C, 30 s at 72 °C; 3 min at 72 °C. PCR products were purified by adding 1 U of Exonuclease I and 0.5 U FastAP Alkaline Phosphatase (Thermo Scientific, St. Leon-Rot, Germany) and incubated at 37 °C for 1 h, followed by inactivation at 80 °C for 15 min. Standard Sanger sequencing was performed in both directions by Macrogen Europe (The Netherlands) with PCR primers, except for *atp*6, for which universal primers M13F-pUC(-40) and M13F(-20) were used; for *tef*1, additional sequencing was performed with two internal primers, EF1-1577F and EF1-1567R (Rehner and Buckley 2005).

### Alignment and phylogeny inference

The sequences were assembled in GENEIOUS Pro v. 6.0.6 (Biomatters) and introns were removed prior to alignment based on the amino acid sequence of previously published sequences. All sequences, including sequences from GenBank, were aligned using MAFFT (Katoh and Standley 2013) on the server accessed at http://mafft.cbrc.jp/alignment/server/.

Maximum Likelihood (ML) phylogenetic inference was performed using RAxML (Stamatakis 2006) on the CIPRES web portal (RAxML-HPC2 on XSEDE; Miller et al. 2009). The phylogenetic tree was inferred by a single analysis with three partitions (one for each gene), using the GTRCAT model with 25 categories, two *Buchwaldoboletus* and nine *Chalciporus* species from sub-family Chalciporoideae were used as outgroup since Chalciporoideae always appeared as sister to the remainder of the Boletaceae in recent phylogenetic analyses (e.g. Nuhn et al. 2013; Wu et al. 2014, 2016). Statistical support of clades was obtained with 1,000 rapid bootstrap replicates.

For Bayesian Inference (BI), the best-fit model of substitution amongst those implementable in MrBayes was estimated separately for each gene using jModeltest (Darriba et al. 2012) on the CIPRES portal, based on the Bayesian Information Criterion (BIC). The selected models were HKY+I+G for *atp*6 and *rpb*2 and GTR+I+G for *cox*3 and *tef*1. Partitioned Bayesian analysis was performed with MrBayes 3.2 (Ronquist et al. 2012) on the CIPRES portal. Two runs of five chains were run for 15,000,000 generations and sampled every 500 generations. The chain temperature was decreased to 0.02 to improve convergence. At the end of the run, the average deviation of split frequencies was 0.008147.

## Results

### Phylogenetic analysis

A total of 325 sequences were newly generated and deposited in GenBank (Table 1). The alignment contained 1,013 sequences from four genes (186 for *atp*6, 358 for *tef*1, 326 for *rpb*2, 143 for *cox*3) from 362 voucher specimens and was 2946 characters long (TreeBase number 23886).

**Figure 1.**
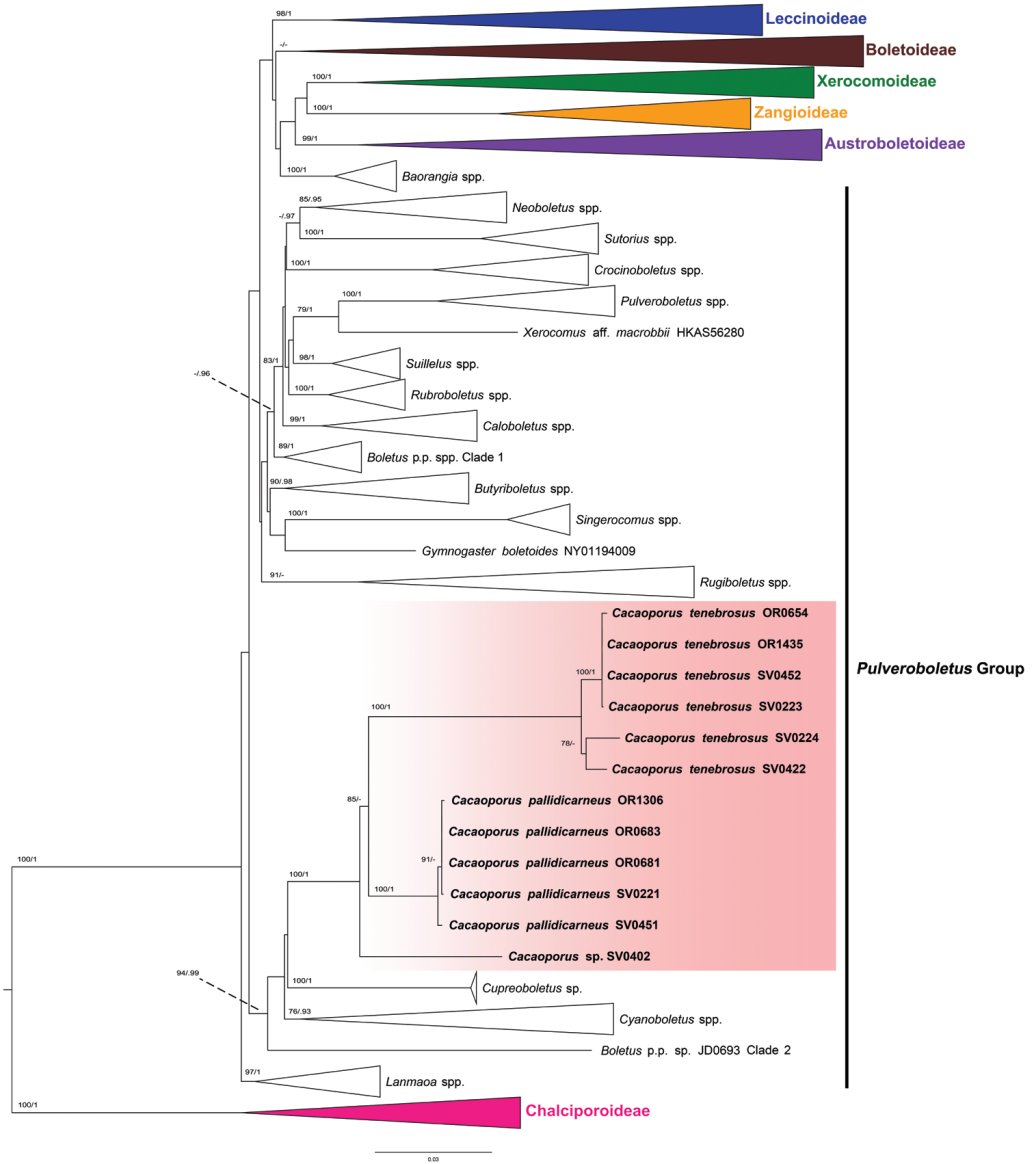
Phylogenetic tree inferred from the four-gene dataset (*atp*6, *cox*3, *rpb*2 and *tef*1), including *Cacaoporus* species and selected Boletaceae using Maximum Likelihood and Bayesian Inference methods (ML tree is presented). The two *Buchwaldoboletus* and nine *Chalciporus* species in subfamily Chalciporoideae were used as outgroup. Most of the taxa not belonging to the *Pulveroboletus* group were collapsed into subfamilies. All genera clades in *Pulveroboletus* group that were highly supported were also collapsed. Bootstrap support values (BS ≥ 70%) and posterior probabilities (PP ≥ 0.90) are shown above the supported branches.

The four-gene analyses retrieved the six subfamilies (Austroboletoideae, Boletoideae, Chalciporoideae, Leccinoideae, Xerocomoideae, Zangioideae) as monophyletic (Fig. 1). The genera belonging to the *Pulveroboletus* group of Wu et al. (2014, 2016) did not form a monophyletic group. The new genus, *Cacaoporus* was monophyletic (BS=100% and PP=1) within a clade containing the genera *Cupreoboletus* Simonini, Gelardi & Vizzini and *Cyanoboletus* Gelardi, Vizzini & Simonini and one undescribed taxon, *Boletus* p.p. sp., clade 2 (specimen voucher JD0693) with high support (BS=94% and PP=0.99). The macromorphologically most similar genus, *Sutorius*, formed another clade (BS=100% and PP=1) sister to *Neoboletus* Gelardi, Simonini & Vizzini, with 67% BS and 0.97 PP support, in another clade of the *Pulveroboletus* group.

Our phylogeny also showed that thirteen *Sutorius* species including *S.brunneissimus* (W.F. Chiu) G. Wu & Zhu L. Yang, *S.ferrugineus* G. Wu, Fang Li & Zhu L. Yang, *S.flavidus* G. Wu & Zhu L. Yang, *S.hainanensis* (T.H. Li & M. Zang) G. Wu & Zhu L. Yang, *S.junquilleus* (Quél.) G. Wu & Zhu L. Yang, *S.magnificus* (W.F. Chiu) G. Wu & Zhu L. Yang, *S.obscureumbrinus* (Hongo) G. Wu & Zhu L. Yang, *S.rubriporus* G. Wu & Zhu L. Yang, *S.sanguineoides* G. Wu & Zhu L. Yang, *S.sanguineus* G. Wu & Zhu L. Yang, *S.tomentulosus* (M. Zang, W.P. Liu & M.R. Hu) G. Wu & Zhu L. Yang and *S.venenatus* (Nagas.) G. Wu & Zhu L. Yang clustered in the *Neoboletus* clade with high support (85% BS and 0.95 PP), while the true *Sutorius*, including the typus generis *S.eximius* (Peck) Halling, Nuhn & Osmundson, formed a different well-supported clade (BS=100% and PP=1).

**Table 1.** List of collections used for DNA analyses, with origin, GenBank accession numbers and reference(s).

Species	Voucher	Origin	*atp*6	*cox*3	*tef*1	*rpb*2	Reference(s)
Afroboletus aff. multijugus	JD671	Burundi	MH614651	MH614794	MH614700	MH614747	This study
* Afroboletus costatisporus *	ADK4644	Togo	KT823958	MH614795*	KT824024	KT823991	Raspé et al. 2016; *This study
* Afroboletus luteolus *	ADK4844	Togo	MH614652	MH614796	MH614701	MH614748	This study
* Aureoboletus catenarius *	HKAS54467	China	–	–	KT990711	KT990349	Wu et al. 2016
* Aureoboletus duplicatoporus *	HKAS50498	China	–	–	KF112230	KF112754	Wu et al. 2014
* Aureoboletus gentilis *	ADK4865	Belgium	KT823961	MH614797*	KT824027	KT823994	Raspé et al. 2016; *This study
* Aureoboletus mirabilis *	HKAS57776	China	–	–	KF112229	KF112743	Wu et al. 2014
* Aureoboletus moravicus *	VDKO1120	Belgium	MG212528	MH614798*	MG212573	MG212615	Vadthanarat et al. 2018; *This study
* Aureoboletus nephrosporus *	HKAS67931	China	–	–	KT990720	KT990357	Wu et al. 2016
* Aureoboletus projectellus *	AFTOL-ID-713	USA	DQ534604*	–	AY879116	AY787218	*Binder and Hibbett 2006; Binder et al., Unpublished
* Aureoboletus shichianus *	HKAS76852	China	–	–	KF112237	KF112756	Wu et al. 2014
*Aureoboletus* sp.	HKAS56317	China	–	–	KF112239	KF112753	Wu et al. 2014
*Aureoboletus* sp.	OR0245	China	MH614653	MH614799	MH614702	MH614749	This study
*Aureoboletus* sp.	OR0369	Thailand	MH614654	MH614800	MH614703	MH614750	This study
* Aureoboletus thibetanus *	HKAS76655	China	–	–	KF112236	KF112752	Wu et al. 2014
* Aureoboletus thibetanus *	AFTOL-ID-450	China	DQ534600*	–	DQ029199	DQ366279	*Binder and Hibbett 2006; Unpublished
* Aureoboletus tomentosus *	HKAS80485	China	–	–	KT990715	KT990353	Wu et al. 2016
* Aureoboletus viscosus *	OR0361	Thailand	MH614655	MH614801	MH614704	MH614751	This study
* Aureoboletus zangii *	HKAS74766	China	–	–	KT990726	KT990363	Wu et al. 2016
Austroboletus cf. dictyotus	OR0045	Thailand	KT823966	MH614802*	KT824032	KT823999	Raspé et al. 2016; *This study
Austroboletus cf. subvirens	OR0573	Thailand	MH614656	MH614803	MH614705	MH614752	This study
* Austroboletus eburneus *	REH9487	Australia	–	–	JX889708	–	Halling et al. 2012b
* Austroboletus olivaceoglutinosus *	HKAS57756	China	–	–	KF112212	KF112764	Wu et al. 2014
*Austroboletus* sp.	HKAS59624	China	–	–	KF112217	KF112765	Wu et al. 2014
*Austroboletus* sp.	OR0891	Thailand	MH614657	MH614804	MH614706	MH614753	This study
* Baorangia major *	OR0209	Thailand	MG897421	MK372295*	MG897431	MG897441	Phookamsak et al. 2019; *This study
* Baorangia pseudocalopus *	HKAS63607	China	–	–	KF112167	KF112677	Wu et al. 2014
* Baorangia pseudocalopus *	HKAS75739	China	–	–	KJ184570	KM605179	Wu et al. 2015
* Baorangia pseudocalopus *	HKAS75081	China	–	–	KF112168	KF112678	Wu et al. 2014
* Baorangia rufomaculata *	BOTH4144	USA	MG897415	MH614805*	MG897425	MG897435	Phookamsak et al. 2019; *This study
* Boletellus ananas *	NY815459	Costa Rica	–	–	KF112308	KF112760	Wu et al. 2014
* Boletellus ananas *	K(M)123769	Belize	MH614658	MH614807	MH614707	MH614754	This study
Boletellus aff. emodensis	OR0061	Thailand	KT823970	MH614806*	KT824036	KT824003	Raspé et al. 2016; *This study
*Boletellus* sp.	HKAS59536	China	–	–	KF112306	KF112758	Wu et al. 2014
*Boletellus* sp.	OR0621	Thailand	MG212529	MH614808*	MG212574	MG212616	Vadthanarat et al. 2018; *This study
* Boletus aereus *	VDKO1055	Belgium	MG212530	MH614809*	MG212575	MG212617	Vadthanarat et al. 2018; *This study
* Boletus albobrunnescens *	OR0131	Thailand	KT823973	MH614810*	KT824039	KT824006	Raspé et al. 2016; *This study
* Boletus botryoides *	HKAS53403	China	–	–	KT990738	KT990375	Wu et al. 2016
* Boletus edulis *	HMJAU4637	Russia	–	–	KF112202	KF112704	Wu et al. 2014
* Boletus edulis *	VDKO0869	Belgium	MG212531	MH614811*	MG212576	MG212618	Vadthanarat et al. 2018; *This study
*Boletus* p.p. sp	JD0693	Burundi	MH645583	–	MH645591	MH645599	This study
*Boletus* p.p. sp.	OR0832	Thailand	MH645584	MH645605	MH645592	MH645600	This study
*Boletus* p.p. sp.	OR1002	Thailand	MH645585	MH645606	MH645593	MH645601	This study
* Boletus pallidus *	BOTH4356	USA	MH614659	MH614812	MH614708	–	This study
* Boletus pallidus *	TDB-1231-Bruns	–	AF002142	AF002154	–	–	Kretzer and Bruns 1999
* Boletus reticuloceps *	HKAS57671	China	–	–	KF112201	KF112703	Wu et al. 2014
*Boletus* s.s. sp.	OR0446	China	MG212532	MH614813*	MG212577	KF112703	Vadthanarat et al. 2018; *This study
*Boletus* sp.	HKAS59660	China	–	–	KF112153	KF112664	Wu et al. 2014
*Boletus* sp.	HKAS63598	China	–	–	KF112152	KF112663	Wu et al. 2014
* Boletus violaceofuscus *	HKAS62900	China	–	–	KF112219	KF112762	Wu et al. 2014
* Borofutus dhakanus *	HKAS73789	Bangladesh	–	–	JQ928576	JQ928597	Hosen et al. 2013
* Borofutus dhakanus *	OR0345	Thailand	MH614660	MH614814	MH614709	MH614755	This study
* Buchwaldoboletus lignicola *	HKAS76674	China	–	–	KF112277	KF112819	Wu et al. 2014
* Buchwaldoboletus lignicola *	VDKO1140	Belgium	MH614661	MH614815	MH614710	MH614756	This study
* Butyriboletus appendiculatus *	VDKO0193b	Belgium	MG212537	MH614816*	MG212582	MG212624	Vadthanarat et al. 2018; *This study
Butyriboletus cf. roseoflavus	OR0230	China	KT823974	MH614819*	KT824040	KT824007	Raspé et al. 2016; *This study
* Butyriboletus frostii *	NY815462	USA	–	–	KF112164	KF112675	Wu et al. 2014
* Butyriboletus pseudoregius *	VDKO0925	Belgium	MG212538	MH614817*	MG212583	MG212625	Vadthanarat et al. 2018; *This study
* Butyriboletus pseudospeciosus *	HKAS63513	China	–	–	KT990743	KT990380	Wu et al. 2016
* Butyriboletus roseoflavus *	HKAS54099	China	–	–	KF739779	KF739703	Wu et al. 2014
* Butyriboletus roseopurpureus *	BOTH4497	USA	MG897418	MH614818*	MG897428	MG897438	Phookamsak et al., 2019; *This study
*Butyriboletus* sp.	HKAS52661	China	–	–	KF112169	KF112676	Wu et al. 2014
*Butyriboletus* sp.	HKAS52525	China	–	–	KF112163	KF112671	Wu et al. 2014
*Butyriboletus* sp.	HKAS57774	China	–	–	KF112155	KF112670	Wu et al. 2014
*Butyriboletus* sp.	HKAS59814	China	–	–	KF112199	KF112699	Wu et al. 2014
*Butyriboletus* sp.	HKAS63528	China	–	–	KF112156	KF112673	Wu et al. 2014
*Butyriboletus* sp.	MHHNU7456	China	–	–	KT990741	KT990378	Wu et al. 2016
* Butyriboletus subsplendidus *	HKAS50444	China	–	–	KT990742	KT990379	Wu et al. 2016
* Butyriboletus yicibus *	HKAS55413	China	–	–	KF112157	KF112674	Wu et al. 2014
* Cacaoporus pallidicarneus *	OR0681	Thailand	MK372259	MK372296	–	MK372283	This study
* Cacaoporus pallidicarneus *	OR0683	Thailand	MK372260	MK372297	–	MK372284	This study
* Cacaoporus pallidicarneus *	OR1306	Thailand	MK372261	MK372298	MK372272	MK372285	This study
* Cacaoporus pallidicarneus *	SV0221	Thailand	MK372262	MK372299	MK372273	MK372286	This study
* Cacaoporus pallidicarneus *	SV0451	Thailand	MK372263	MK372300	MK372274	MK372287	This study
*Cacaoporus* sp.	SV0402	Thailand	MK372270	–	MK372281	MK372293	This study
* Cacaoporus tenebrosus *	OR0654	Thailand	MK372264	MK372301	MK372275	MK372288	This study
* Cacaoporus tenebrosus *	OR1435	Thailand	MK372265	MK372302	MK372276	MK372289	This study
* Cacaoporus tenebrosus *	SV0223	Thailand	MK372266	MK372303	MK372277	MK372290	This study
* Cacaoporus tenebrosus *	SV0224	Thailand	MK372267	MK372304	MK372278	MK372291	This study
* Cacaoporus tenebrosus *	SV0422	Thailand	MK372268	MK372305	MK372279	–	This study
* Cacaoporus tenebrosus *	SV0452	Thailand	MK372269	MK372306	MK372280	MK372292	This study
Caloboletus aff. calopus	HKAS74739	China	–	–	KF112166	KF112667	Wu et al. 2014
* Caloboletus calopus *	ADK4087	Belgium	MG212539	MH614820	KJ184566	KP055030	Vadthanarat et al. 2018; Zhao et al. 2014a, b; This study
* Caloboletus inedulis *	BOTH3963	USA	MG897414	MH614821*	MG897424	MG897434	Phookamsak et al. 2019; *This study
* Caloboletus panniformis *	HKAS55444	China	–	–	KF112165	KF112666	Wu et al. 2014
* Caloboletus radicans *	VDKO1187	Belgium	MG212540	MH614822*	MG212584	MG212626	Vadthanarat et al. 2018; *This study
*Caloboletus* sp.	HKAS53353	China	–	–	KF112188	KF112668	Wu et al. 2014
*Caloboletus* sp.	OR0068	Thailand	MH614662	MH614823	MH614711	MH614757	This study
* Caloboletus yunnanensis *	HKAS69214	China	–	–	KJ184568	KT990396	Zhao et al. 2014a; Wu et al. 2016
Chalciporus aff. piperatus	OR0586	Thailand	KT823976	MH614824*	KT824042	KT824009	Raspé et al. 2016; *This study
Chalciporus aff. rubinus	OR0139	China	MH614663	–	MH614712	MH614758	This study
* Chalciporus africanus *	JD517	Cameroon	KT823963	MH614825*	KT824029	KT823996	Raspé et al. 2016; *This study
* Chalciporus piperatus *	VDKO1063	Belgium	MH614664	MH614826	MH614713	MH614759	This study
* Chalciporus rubinus *	AF2835	Belgium	KT823962	–	KT824028	KT823995	Raspé et al. 2016
*Chalciporus* sp.	HKAS53400	China	–	–	KF112279	KF112821	Wu et al. 2014
*Chalciporus* sp.	HKAS74779	China	–	–	KF112278	KF112820	Wu et al. 2014
*Chalciporus* sp.	OR0363	Thailand	MH645586	MH645607	MH645594	MH645602	This study
*Chalciporus* sp.	OR0373	Thailand	MH645587	MH645608	MH645595	MH645603	This study
*Chiua* sp.	OR0141	China	MH614665	MH614827	MH614714	MH614760	This study
* Chiua virens *	OR0266	China	MG212541	MH614828*	MG212585	MG212627	Vadthanarat et al. 2018; *This study
* Chiua viridula *	HKAS74928	China	–	–	KF112273	KF112794	Wu et al. 2014
Crocinoboletus cf. laetissimus	OR0576	Thailand	KT823975	MH614833*	KT824041	KT824008	Raspé et al. 2016; *This study
* Crocinoboletus rufoaureus *	HKAS53424	China	–	–	KF112206	KF112710	Wu et al. 2014
* Cupreoboletus poikilochromus *	GS10070	Italy	–	–	KT157072	KT157068	Gelardi et al. 2015
* Cupreoboletus poikilochromus *	GS11008	Italy	–	–	KT157071	KT157067	Gelardi et al. 2015
* Cyanoboletus brunneoruber *	HKAS80579_1	China	–	–	KT990763	KT990401	Wu et al. 2016
* Cyanoboletus brunneoruber *	OR0233	China	MG212542	MH614834*	MG212586	MG212628	Vadthanarat et al. 2018; *This study
* Cyanoboletus instabilis *	HKAS59554	China	–	–	KF112186	KF112698	Wu et al. 2014
* Cyanoboletus pulverulentus *	RW109	Belgium	KT823980	MH614835*	KT824046	KT824013	Raspé et al. 2016; *This study
* Cyanoboletus sinopulverulentus *	HKAS59609	China	–	–	KF112193	KF112700	Wu et al. 2014
*Cyanoboletus* sp.	HKAS52639	China	–	–	KF112195	KF112701	Wu et al. 2014
*Cyanoboletus* sp.	HKAS76850	China	–	–	KF112187	KF112697	Wu et al. 2014
*Cyanoboletus* sp.	OR0257	China	MG212543	MH614836*	MG212587	MG212629	Vadthanarat et al. 2018; *This study
*Cyanoboletus* sp.	HKAS90208_1	China	–	–	KT990766	KT990404	Wu et al. 2016
*Cyanoboletus* sp.	OR0322	Thailand	MH614673	MH614837	MH614722	MH614768	This study
*Cyanoboletus* sp.	OR0491	China	MH614674	MH614838	MH614723	MH614769	This study
*Cyanoboletus* sp.	OR0961	Thailand	MH614675	MH614839	MH614724	MH614770	This study
* Fistulinella prunicolor *	REH9880	Australia	MH614676	MH614840	MH614725	MH614771	This study
* Gymnogaster boletoides *	NY01194009	Australia	–	–	KT990768	KT990406	Wu et al. 2016
* Harrya atriceps *	REH7403	Costa Rica	–	–	JX889702	–	Halling et al. 2012b
* Harrya chromapes *	HKAS50527	China	–	–	KF112270	KF112792	Wu et al. 2014
* Harrya moniliformis *	HKAS49627	China	–	–	KT990881	KT990500	Wu et al. 2016
Heimioporus cf. mandarinus	OR0661	Thailand	MG212545	MH614841*	MG212589	MG212631	Vadthanarat et al. 2018; *This study
* Heimioporus japonicus *	OR0114	Thailand	KT823971	MH614842*	KT824037	KT824004	Raspé et al. 2016; *This study
* Heimioporus retisporus *	HKAS52237	China	–	–	KF112228	KF112806	Wu et al. 2014
*Heimioporus* sp.	OR0218	Thailand	MG212546	–	MG212590	MG212632	Vadthanarat et al. 2018
* Hemileccinum depilatum *	AF2845	Belgium	MG212547	MH614843*	MG212591	MG212633	Vadthanarat et al. 2018; *This study
* Hemileccinum impolitum *	ADK4078	Belgium	MG212548	MH614844*	MG212592	MG212634	Vadthanarat et al. 2018; *This study
* Hemileccinum indecorum *	OR0863	Thailand	MH614677	MH614845	MH614726	MH614772	This study
* Hemileccinum rugosum *	HKAS84970	China	–	–	KT990773	KT990412	Wu et al. 2016
* Hortiboletus amygdalinus *	HKAS54166	China	–	–	KT990777	KT990416	Wu et al. 2016
* Hortiboletus rubellus *	VDKO0403	Belgium	MH614679	MH614847	–	MH614774	This study
*Hortiboletus* sp.	HKAS50466	China	–	–	KF112183	KF112694	Wu et al. 2014
*Hortiboletus* sp.	HKAS51239	China	–	–	KF112184	KF112695	Wu et al. 2014
*Hortiboletus* sp.	HKAS51292	China	–	–	KF112181	KF112692	Wu et al. 2014
*Hortiboletus* sp.	HKAS76673	China	–	–	KF112182	KF112693	Wu et al. 2014
* Hortiboletus subpaludosus *	HKAS59608	China	–	–	KF112185	KF112696	Wu et al. 2014
Hourangia cf. pumila	OR0762	Thailand	MH614680	MH614848	MH614728	MH614775	This study
* Hourangia cheoi *	HKAS74744	China	–	–	KF112285	KF112772	Wu et al. 2014
* Hourangia cheoi *	Zhu108	China	–	–	KP136979	KP136928	Zhu et al. 2015
* Hourangia nigropunctata *	HKAS 57427	China	–	–	KP136927	KP136978	Zhu et al. 2015
* Hymenoboletus luteopurpureus *	HKAS46334	China	–	–	KF112271	KF112795	Wu et al. 2014
* Imleria badia *	VDKO0709	Belgium	KT823983	MH614849*	KT824049	KT824016	Raspé et al. 2016; *This study
* Imleria obscurebrunnea *	OR0263	China	MH614681	MH614850	MH614729	MH614776	This study
* Imleria subalpina *	HKAS74712	China	–	–	KF112189	KF112706	Wu et al. 2014
* Lanmaoa angustispora *	HKAS74759	China	–	–	KM605155	KM605178	Wu et al. 2015
* Lanmaoa angustispora *	HKAS74765	China	–	–	KF112159	KF112680	Wu et al. 2014
* Lanmaoa angustispora *	HKAS74752	China	–	–	KM605154	KM605177	Wu et al. 2015
* Lanmaoa asiatica *	HKAS54094	China	–	–	KF112161	KF112682	Wu et al. 2014
* Lanmaoa asiatica *	HKAS63516	China	–	–	KT990780	KT990419	Wu et al. 2016
* Lanmaoa asiatica *	OR0228	China	MH614682	MH614851	MH614730	MH614777	This study
* Lanmaoa carminipes *	BOTH4591	USA	MG897419	MH614852*	MG897429	MG897439	Phookamsak et al. 2019, *This study
* Lanmaoa flavorubra *	NY775777	Costa Rica	–	–	KF112160	KF112681	Wu et al. 2014
* Lanmaoa pallidorosea *	BOTH4432	USA	MG897417	MH614853*	MG897427	MG897437	Phookamsak et al. 2019, *This study
*Lanmaoa* sp.	HKAS52518	China	–	–	KF112162	KF112683	Wu et al. 2014
*Lanmaoa* sp.	OR0130	Thailand	MH614683	MH614854	MH614731	MH614778	This study
*Lanmaoa* sp.	OR0370	Thailand	MH614684	MH614855	MH614732	MH614779	This study
Leccinellum aff. crocipodium	HKAS76658	China	–	–	KF112252	KF112728	Wu et al. 2014
Leccinellum aff. griseum	KPM-NC-0017832	Japan	KC552164	–	JN378450*	–	unpublished, *Orihara et al. 2012
* Leccinellum corsicum *	Buf4507	USA	–	–	KF030435	–	Nuhn et al. 2013
* Leccinellum cremeum *	HKAS90639	China	–	–	KT990781	KT990420	Wu et al. 2016
* Leccinellum crocipodium *	VDKO1006	Belgium	KT823988	MH614856*	KT824054	KT824021	Raspé et al. 2016; *This study
*Leccinellum* sp.	KPM-NC-0018041	Japan	KC552165	–	KC552094	–	Orihara et al. 2016
*Leccinellum* sp.	OR0711	Thailand	MH614685	–	MH614733	MH614780	This study
* Leccinum monticola *	HKAS76669	China	–	–	KF112249	KF112723	Wu et al. 2014
* Leccinum quercinum *	HKAS63502	China	–	–	KF112250	KF112724	Wu et al. 2014
* Leccinum scabrum *	RW105a	Belgium	KT823979	MH614857*	KT824045	KT824012	Raspé et al. 2016; *This study
* Leccinum scabrum *	VDKO0938	Belgium	MG212549	MH614858*	MG212593	MG212635	Vadthanarat et al. 2018; *This study
* Leccinum scabrum *	KPM-NC-0017840	Scotland	KC552170	–	JN378455	–	Orihara et al. 2016, 2012
* Leccinum schistophilum *	VDKO1128	Belgium	KT823989	MH614859*	KT824055	KT824022	Raspé et al. 2016; *This study
* Leccinum variicolor *	VDKO0844	Belgium	MG212550	MH614860*	MG212594	MG212636	Vadthanarat et al. 2018; *This study
* Mucilopilus castaneiceps *	HKAS75045	China	–	–	KF112211	KF112735	Wu et al. 2014
* Neoboletus brunneissimus *	HKAS50538	China	–	–	KM605150	KM605173	Wu et al. 2015
* Neoboletus brunneissimus *	HKAS52660	China	–	–	KF112143	KF112650	Wu et al. 2014
* Neoboletus brunneissimus *	HKAS57451	China	–	–	KM605149	KM605172	Wu et al. 2015
* Neoboletus brunneissimus *	OR0249	China	MG212551	MH614861*	MG212595	MG212637	Vadthanarat et al. 2018; *This study
* Neoboletus erythropus *	VDKO0690	Belgium	KT823982	MH614864*	KT824048	KT824015	Raspé et al. 2016; *This study
* Neoboletus ferrugineus *	HKAS77718	China	–	–	KT990789	KT990431	Wu et al. 2016
* Neoboletus ferrugineus *	HKAS77617	China	–	–	KT990788	KT990430	Wu et al. 2016
* Neoboletus flavidus *	HKAS59443	China	–	–	KU974136	KU974144	Wu et al. 2016
* Neoboletus flavidus *	HKAS58724	China	–	–	KU974137	KU974145	Wu et al. 2016
* Neoboletus hainanensis *	HKAS63515	China	–	–	KT990808	KT990449	Wu et al. 2016
* Neoboletus hainanensis *	HKAS74880	China	–	–	KT990790	KT990432	Wu et al. 2016
* Neoboletus hainanensis *	HKAS90209	China	–	–	KT990809	KT990450	Wu et al. 2016
* Neoboletus hainanensis *	HKAS59469	China	–	–	KF112175	KF112669	Wu et al. 2014
* Neoboletus junquilleus *	AF2922	France	MG212552	MH614862*	MG212596	MG212638	Vadthanarat et al. 2018; *This study
* Neoboletus magnificus *	HKAS54096	China	–	–	KF112149	KF112654	Wu et al. 2014
* Neoboletus magnificus *	HKAS74939	China	–	–	KF112148	KF112653	Wu et al. 2014
* Neoboletus multipunctatus *	HKAS76851	China	–	–	KF112144	KF112651	Wu et al. 2014
* Neoboletus multipunctatus *	OR0128	Thailand	MH614686	MH614863	MH614734	MH614781	This study
* Neoboletus obscureumbrinus *	OR0553	Thailand	MK372271	–	MK372282	MK372294	This study
* Neoboletus obscureumbrinus *	HKAS63498	China	–	–	KT990791	KT990433	Wu et al. 2016
* Neoboletus obscureumbrinus *	HKAS77774	China	–	–	KT990792	KT990434	Wu et al. 2016
* Neoboletus obscureumbrinus *	HKAS89014	China	–	–	KT990793	KT990435	Wu et al. 2016
* Neoboletus obscureumbrinus *	HKAS89027	China	–	–	KT990794	KT990436	Wu et al. 2016
* Neoboletus rubriporus *	HKAS57512	China	–	–	KF112151	KF112656	Wu et al. 2014
* Neoboletus rubriporus *	HKAS83026	China	–	–	KT990795	KT990437	Wu et al. 2016
* Neoboletus sanguineoides *	HKAS57766	China	–	–	KT990799	KT990440	Wu et al. 2016
* Neoboletus sanguineoides *	HKAS74733	China	–	–	KT990800	KT990441	Wu et al. 2016
* Neoboletus sanguineoides *	HKAS55440	China	–	–	KF112145	KF112652	Wu et al. 2014
* Neoboletus sanguineus *	HKAS80823	China	–	–	KT990802	KT990442	Wu et al. 2016
* Neoboletus tomentulosus *	HKAS77656	China	–	–	KT990806	KT990446	Wu et al. 2016
* Neoboletus tomentulosus *	HKAS53369	China	–	–	KF112154	KF112659	Wu et al. 2014
* Neoboletus venenatus *	HKAS57489	China	–	–	KF112158	KF112665	Wu et al. 2014
* Neoboletus venenatus *	HKAS63535	China	–	–	KT990807	KT990448	Wu et al. 2016
*Neoboletus* sp.	HKAS76660	China	–	–	KF112180	KF112731	Wu et al. 2014
* Octaviania asahimontana *	KPM-NC-17824	Japan	KC552154	–	JN378430	–	Orihara et al. 2016, 2012
* Octaviania asterosperma *	AQUI3899	Italy	KC552159	–	KC552093	–	Orihara et al. 2016
* Octaviania celatifilia *	KPM-NC-17776	Japan	KC552147	–	JN378416	–	Orihara et al. 2016, 2012
* Octaviania cyanescens *	PNW-FUNGI-5603	USA	KC552160	–	JN378438	–	Orihara et al. 2016, 2012
* Octaviania decimae *	KPM-NC17763	Japan	KC552145	–	JN378409	–	Orihara et al. 2016, 2012
* Octaviania tasmanica *	MEL2128484	Australia	KC552157	–	JN378437	–	Orihara et al. 2016, 2012
* Octaviania tasmanica *	MEL2341996	Australia	KC552156	–	JN378436	–	Orihara et al. 2016, 2012
* Octaviania zelleri *	MES270	USA	KC552161	–	JN378440	–	Orihara et al. 2016, 2012
* Parvixerocomus pseudoaokii *	OR0155	China	MG212553	MH614865	MG212597	MG212639	This study
* Phylloporus bellus *	OR0473	China	MH580778	MH614866*	MH580798	MH580818	Chuankid et al. 2019; *This study
* Phylloporus brunneiceps *	OR0050	Thailand	KT823968	MH614867*	KT824034	KT824001	Raspé et al. 2016; *This study
* Phylloporus castanopsidis *	OR0052	Thailand	KT823969	MH614868*	KT824035	KT824002	Raspé et al. 2016; *This study
* Phylloporus imbricatus *	HKAS68642	China	–	–	KF112299	KF112786	Wu et al. 2014
* Phylloporus luxiensis *	HKAS75077	China	–	–	KF112298	KF112785	Wu et al. 2014
* Phylloporus maculatus *	OR0285	China	MH580780	–	MH580800	MH580820	Chuankid et al. 2019
* Phylloporus pelletieri *	WU18746	Austria	MH580781	MH614869*	MH580801	MH580821	Chuankid et al. 2019; *This study
* Phylloporus pusillus *	OR1158	Thailand	MH580783	MH614870*	MH580803	MH580823	Chuankid et al. 2019; *This study
* Phylloporus rhodoxanthus *	WU17978	USA	MH580785	MH614871*	MH580805	MH580824	Chuankid et al. 2019; *This study
* Phylloporus rubeolus *	OR0251	China	MH580786	MH614872*	MH580806	MH580825	Chuankid et al. 2019; *This study
* Phylloporus rubiginosus *	OR0169	China	MH580788	MH614873*	MH580808	MH580827	Chuankid et al. 2019; *This study
*Phylloporus* sp.	OR0896	Thailand	MH580790	MH614874*	MH580810	MH580829	Chuankid et al. 2019; *This study
* Phylloporus subbacillisporus *	OR0436	China	MH580792	MH614875*	MH580812	MH580831	Chuankid et al. 2019; *This study
* Phylloporus subrubeolus *	BC022	Thailand	MH580793	MH614876*	MH580813	MH580832	Chuankid et al. 2019; *This study
* Phylloporus yunnanensis *	OR0448	China	MG212554	MH614877*	MG212598	MG212640	Vadthanarat et al. 2018; *This study
* Porphyrellus castaneus *	OR0241	China	MG212555	MH614878*	MG212599	MG212641	Vadthanarat et al. 2018; *This study
Porphyrellus cf. nigropurpureus	ADK3733	Benin	MH614687	MH614879	MH614735	MH614782	This study
* Porphyrellus nigropurpureus *	HKAS74938	China	–	–	KF112246	KF112763	Wu et al. 2014
* Porphyrellus porphyrosporus *	MB97 023	Germany	DQ534609	–	GU187734	GU187800	Binder and Hibbett 2006; Binder et al. 2010
*Porphyrellus* sp.	HKAS53366	China	–	–	KF112241	KF112716	Wu et al. 2014
*Porphyrellus* sp.	JD659	Burundi	MH614688	MH614880	MH614736	MH614783	This study
*Porphyrellus* sp.	OR0222	Thailand	MH614689	MH614881	MH614737	MH614784	This study
Pulveroboletus aff. ravenelii	HKAS50203	China	–	–	KT990810	KT990451	Wu et al. 2016
Pulveroboletus aff. ravenelii	ADK4360	Togo	KT823957	MH614882*	KT824023	KT823990	Raspé et al. 2016; *This study
Pulveroboletus aff. ravenelii	ADK4650	Togo	KT823959	MH614883*	KT824025	KT823992	Raspé et al. 2016; *This study
Pulveroboletus aff. ravenelii	HKAS53351	China	–	–	KF112261	KF112712	Wu et al. 2014
* Pulveroboletus brunneopunctatus *	HKAS52615	China	–	–	KT990813	KT990454	Wu et al. 2016
* Pulveroboletus brunneopunctatus *	HKAS55369	China	–	–	KT990814	KT990455	Wu et al. 2016
* Pulveroboletus brunneopunctatus *	HKAS74926	China	–	–	KT990815	KT990456	Wu et al. 2016
* Pulveroboletus fragrans *	OR0673	Thailand	KT823977	MH614884*	KT824043	KT824010	Raspé et al. 2016; *This study
* Pulveroboletus macrosporus *	HKAS57628	China	–	–	KT990812	KT990453	Wu et al. 2016
* Pulveroboletus ravenelii *	REH2565	USA	KU665635	MH614885*	KU665636	KU665637	Raspé et al. 2016; *This study
*Pulveroboletus* sp.	HKAS74933	China	–	–	KF112262	KF112713	Wu et al. 2014
*Pulveroboletus* sp.	HKAS57665	China	–	–	KF112264	KF112715	Wu et al. 2014
Retiboletus aff. nigerrimus	OR0049	Thailand	KT823967	MH614886*	KT824033	KT824000	Raspé et al. 2016; *This study
* Retiboletus brunneolus *	HKAS52680	China	–	–	KF112179	KF112690	Wu et al. 2014
* Retiboletus fuscus *	HKAS59460	China	–	–	JQ928580	JQ928601	Hosen et al. 2013
* Retiboletus fuscus *	OR0231	China	MG212556	MH614887*	MG212600	MG212642	Vadthanarat et al. 2018; *This study
* Retiboletus fuscus *	HKAS63624	China	–	–	KT990829	KT990466	Wu et al. 2016
* Retiboletus fuscus *	HKAS74756	China	–	–	KT990830	KT990467	Wu et al. 2016
* Retiboletus griseus *	MB03 079	USA	KT823964	MH614888*	KT824030	KT823997	Raspé et al. 2016; *This study
* Retiboletus griseus *	HKAS63590	China	–	–	KF112178	KF112691	Wu et al. 2014
* Retiboletus kauffmanii *	OR0278	China	MG212557	MH614889*	MG212601	MG212643	Vadthanarat et al. 2018; *This study
* Retiboletus nigerrimus *	HKAS53418	China	–	–	KT990824	KT990462	Wu et al. 2016
* Retiboletus sinensis *	HKAS59832	China	–	–	KT990827	KT990464	Wu et al. 2016
* Retiboletus zhangfeii *	HKAS59699	China	–	–	JQ928582	JQ928603	Hosen et al. 2013
* Rhodactina himalayensis *	CMU25117	Thailand	MG212558	–	MG212602, MG212603	–	Vadthanarat et al. 2018
* Rhodactina rostratispora *	SV170	Thailand	MG212560	–	MG212605	MG212645	Vadthanarat et al. 2018
* Rossbeevera cryptocyanea *	KPM-NC17843	Japan	KT581441	–	KC552072	–	Orihara et al. 2016
* Rossbeevera eucyanea *	TNS-F-36986	Japan	KC552115	–	KC552068	–	Orihara et al. 2016
* Rossbeevera griseovelutina *	TNS-F-36989	Japan	KC552124	–	KC552076	–	Orihara et al. 2016
* Rossbeevera pachydermis *	KPM-NC23336	New Zealand	KJ001064	–	KP222912	–	Orihara et al. 2016
* Rossbeevera vittatispora *	OSC61484	Australia	KC552109	–	JN378446	–	Orihara et al. 2016, 2012
* Royoungia reticulata *	HKAS52253	China	–	–	KT990786	KT990427	Wu et al. 2016
* Royoungia rubina *	HKAS53379	China	–	–	KF112274	KF112796	Wu et al. 2014
* Rubroboletus latisporus *	HKAS80358	China	–	–	KP055020	KP055029	Zhao et al. 2014b
* Rubroboletus legaliae *	VDKO0936	Belgium	KT823985	MH614890*	KT824051	KT824018	Raspé et al. 2016; *This study
* Rubroboletus rhodosanguineus *	BOTH4263	USA	MG897416	MH614891*	MG897426	MG897436	Phookamsak et al. 2019, *This study
* Rubroboletus rhodoxanthus *	HKAS84879	Germany	–	–	KT990831	KT990468	Wu et al. 2016
* Rubroboletus satanas *	VDKO0968	Belgium	KT823986	MH614892*	KT824052	KT824019	Raspé et al. 2016; *This study
* Rubroboletus sinicus *	HKAS68620	China	–	–	KF112146	KF112661	Wu et al. 2014
* Rubroboletus sinicus *	HKAS56304	China	–	–	KJ619483	KP055031	Zhao et al. 2014a; Zhao et al. 2014b
*Rubroboletus* sp.	HKAS68679	China	–	–	KF112147	KF112662	Wu et al. 2014
* Rugiboletus brunneiporus *	HKAS68586	China	–	–	KF112197	KF112719	Wu et al. 2014
* Rugiboletus brunneiporus *	HKAS83009	China	–	–	KM605146	KM605169	Wu et al. 2015
* Rugiboletus brunneiporus *	HKAS83209	China	–	–	KM605144	KM605168	Wu et al. 2015
* Rugiboletus extremiorientalis *	HKAS76663	China	–	–	KM605147	KM605170	Wu et al. 2015
* Rugiboletus extremiorientalis *	OR0406	Thailand	MG212562	MH614893*	MG212607	MG212647	Vadthanarat et al. 2018; *This study
*Rugiboletus* sp.	HKAS55373	China	–	–	KF112303	KF112804	Wu et al. 2014
* Singerocomus inundabilis *	TWH9199	Guyana	MH645588	MH645609	MH645596	LC043089*	*Henkel et al. 2016; This study
* Singerocomus rubriflavus *	TWH9585	Guyana	MH645589	MH645610	MH645597	–	This study
* Spongiforma thailandica *	DED7873	Thailand	MG212563	MH614894**	KF030436*	MG212648	*Nuhn et al. 2013; Vadthanarat et al. 2018; **This study
* Strobilomyces atrosquamosus *	HKAS55368	China	–	–	KT990839	KT990476	Wu et al. 2016
* Strobilomyces echinocephalus *	OR0243	China	MG212564	–	MG212608	MG212649	Vadthanarat et al. 2018
* Strobilomyces mirandus *	OR0115	Thailand	KT823972	MH614896*	KT824038	KT824005	Raspé et al. 2016; *This study
* Strobilomyces strobilaceus *	MB03 102	USA	DQ534607*	–	AY883428	AY786065	*Binder and Hibbett 2006, Unpublished
* Strobilomyces strobilaceus *	RW103	Belgium	KT823978	MH614895*	KT824044	KT824011	Raspé et al. 2016; *This study
* Strobilomyces verruculosus *	HKAS55389	China	–	–	KF112259	KF112813	Wu et al. 2014
*Strobilomyces* sp.	OR0259	China	MG212565	MH614897*	MG212609	MG212650	Vadthanarat et al. 2018; *This study
*Strobilomyces* sp.	OR0319	Thailand	MH614690	MH614898	MH614738	MH614785	This study
*Strobilomyces* sp.	OR0778	Thailand	MG212566	MH614899*	MG212610	MG212651	Vadthanarat et al. 2018; *This study
*Strobilomyces* sp.	OR1092	Thailand	MH614691	MH614900	MH614739	MH614786	This study
* Suillellus amygdalinus *	112605ba	USA	–	–	JQ327024	–	Halling et al. 2012a
* Suillellus luridus *	VDKO0241b	Belgium	KT823981	MH614901*	KT824047	KT824014	Raspé et al. 2016; *This study
* Suillellus queletii *	VDKO1185	Belgium	MH645590	MH645611	MH645598	MH645604	This study
* Suillellus subamygdalinus *	HKAS57262	China	–	–	KF112174	KF112660	Wu et al. 2014
* Suillellus subamygdalinus *	HKAS53641	China	–	–	KT990841	KT990478	Wu et al. 2016
* Suillellus subamygdalinus *	HKAS74745	China	–	–	KT990843	KT990479	Wu et al. 2016
Sutorius aff. eximius	HKAS52672	China	–	–	KF112207	KF112802	Wu et al. 2014
Sutorius aff. eximius	HKAS56291	China	–	–	KF112208	KF112803	Wu et al. 2014
* Sutorius australiensis *	REH9441	Australia	MG212567	MK386576**	JQ327032*	MG212652	*Halling et al. 2012a; Vadthanarat et al. 2018; **This study
* Sutorius eximius *	HKAS59657	China	–	–	KT990887	KT990505	Wu et al. 2016
* Sutorius eximius *	REH9400	USA	MG212568	MH614902**	JQ327029*	MG212653	*Halling et al. 2012a; Vadthanarat et al. 2018; **This study
* Sutorius eximius *	HKAS50420	China	–	–	KT990750	KT990387	Wu et al. 2016
*Sutorius* sp.	OR0378B	Thailand	MH614692	MH614903	MH614740	MH614787	This study
*Sutorius* sp.	OR0379	Thailand	MH614693	MH614904	MH614741	MH614788	This study
* Tengioboletus glutinosus *	HKAS53425	China	–	–	KF112204	KF112800	Wu et al. 2014
* Tengioboletus reticulatus *	HKAS53426	China	–	–	KF112313	KF112828	Wu et al. 2014
*Tengioboletus* sp.	HKAS76661	China	–	–	KF112205	KF112801	Wu et al. 2014
* Turmalinea persicina *	KPM-NC18001	Japan	KC552130	–	KC552082	–	Orihara et al. 2016
* Turmalinea yuwanensis *	KPM-NC18011	Japan	KC552138	–	KC552089	–	Orihara et al. 2016
* Tylocinum griseolum *	HKAS50281	China	–	–	KF112284	KF112730	Wu et al. 2014
* Tylopilus alpinus *	HKAS55438	China	–	–	KF112191	KF112687	Wu et al. 2014
* Tylopilus atripurpureus *	HKAS50208	China	–	–	KF112283	KF112799	Wu et al. 2014
*Tylopilusballoui* s.l.	OR0039	Thailand	KT823965	MH614905*	KT824031	KT823998	Raspé et al. 2016; *This study
* Tylopilus brunneirubens *	HKAS53388	China	–	–	KF112192	KF112688	Wu et al. 2014
* Tylopilus felleus *	VDKO0992	Belgium	KT823987	MH614906*	KT824053	KT824020	Raspé et al. 2016; *This study
* Tylopilus ferrugineus *	BOTH3639	USA	MH614694	MH614907	MH614742	MH614789	This study
* Tylopilus otsuensis *	HKAS53401	China	–	–	KF112224	KF112797	Wu et al. 2014
*Tylopilus* sp.	HKAS74925	China	–	–	KF112222	KF112739	Wu et al. 2014
*Tylopilus* sp.	HKAS50229	China	–	–	KF112216	KF112769	Wu et al. 2014
*Tylopilus* sp.	JD598	Gabon	MH614695	MH614908	MH614743	MH614790	This study
*Tylopilus* sp.	OR0252	China	MG212569	MH614909*	MG212611	MG212654	Vadthanarat et al. 2018; *This study
*Tylopilus* sp.	OR0542	Thailand	MG212570	MH614910*	MG212612	MG212655	Vadthanarat et al. 2018; *This study
*Tylopilus* sp.	OR0583	Thailand	MH614696	–	MH614744	–	This study
*Tylopilus* sp.	OR1009	Thailand	MH614697	MH614911	–	MH614791	This study
* Tylopilus vinaceipallidus *	HKAS50210	China	–	–	KF112221	KF112738	Wu et al. 2014
* Tylopilus vinaceipallidus *	OR0137	China	MG212571	MH614912*	MG212613	MG212656	Vadthanarat et al. 2018; *This study
* Tylopilus violaceobrunneus *	HKAS89443	China	–	–	KT990886	KT990504	Wu et al. 2016
* Tylopilus virens *	KPM-NC-0018054	Japan	KC552174	–	KC552103	–	Unpublished
* Veloporphyrellus alpinus *	HKAS68301	China	JX984515	–	JX984550	–	Li et al. 2014b
* Veloporphyrellus conicus *	REH8510	Belize	MH614698	MH614913	MH614745	MH614792	This study
* Veloporphyrellus gracilioides *	HKAS53590	China	–	–	KF112210	KF112734	Wu et al. 2014
* Veloporphyrellus pseudovelatus *	HKAS59444	China	JX984519	–	JX984553	–	Li et al. 2014b
* Veloporphyrellus velatus *	HKAS63668	China	JX984523	–	JX984554	–	Li et al. 2014b
* Xanthoconium affine *	NY00815399	USA	–	–	KT990850	KT990486	Wu et al. 2016
* Xanthoconium porophyllum *	HKAS90217	China	–	–	KT990851	KT990487	Wu et al. 2016
* Xanthoconium sinense *	HKAS77651	China	–	–	KT990853	KT990488	Wu et al. 2016
* Xerocomellus chrysenteron *	VDKO0821	Belgium	KT823984	MH614914*	KT824050	KT824017	Raspé et al. 2016; *This study
* Xerocomellus cisalpinus *	ADK4864	Belgium	KT823960	MH614915*	KT824026	KT823993	Raspé et al. 2016; *This study
* Xerocomellus communis *	HKAS50467	China	–	–	KT990858	KT990494	Wu et al. 2016
* Xerocomellus corneri *	HKAS90206	Philippines	–	–	KT990857	KT990493	Wu et al. 2016
* Xerocomellus porosporus *	VDKO0311	Belgium	MH614678	MH614846	MH614727	MH614773	This study
* Xerocomellus ripariellus *	VDKO0404	Belgium	MH614699	MH614916	MH614746	MH614793	This study
*Xerocomellus* sp.	HKAS56311	China	–	–	KF112170	KF112684	Wu et al. 2014
Xerocomus aff. macrobbii	HKAS56280	China	–	–	KF112265	KF112708	Wu et al. 2014
* Xerocomus fulvipes *	HKAS76666	China	–	–	KF112292	KF112789	Wu et al. 2014
* Xerocomus magniporus *	HKAS58000	China	–	–	KF112293	KF112781	Wu et al. 2014
*Xerocomus* s.s. sp.	OR0237	China	MH580796	–	MH580816	MH580835	Chuankid et al. 2019
*Xerocomus* s.s. sp.	OR0443	China	MH580797	MH614917*	MH580817	MH580836	Chuankid et al. 2019; *This study
*Xerocomus* sp.	OR0053	Thailand	MH580795	MH614918*	MH580815	MH580834	Chuankid et al. 2019; *This study
* Xerocomus subtomentosus *	VDKO0987	Belgium	MG212572	MH614919*	MG212614	MG212657	Vadthanarat et al. 2018; *This study
* Zangia citrina *	HKAS52684	China	HQ326850	–	HQ326872	–	Li et al. 2011
* Zangia olivacea *	HKAS45445	China	HQ326854	–	HQ326873	–	Li et al. 2011
* Zangia olivaceobrunnea *	HKAS52272	China	HQ326857	–	HQ326876	–	Li et al. 2011
* Zangia roseola *	HKAS51137	China	HQ326858	–	HQ326877	–	Li et al. 2011
* Zangia roseola *	HKAS75046	China	–	–	KF112269	KF112791	Wu et al. 2014

## Taxonomy

### Cacaoporus

Taxon classificationFungiBoletalesBoletaceae

Raspé & Vadthanarat gen. nov.

MB829655

#### Etymology.

Refers to the dark, chocolate brown hymenophore and overall colour of basidiomata.

#### Diagnosis.

Similar to the genus *Sutorius* in having brown basidiomata with brown encrustations in the flesh but differs from *Sutorius* in having the following combination of characters: brown to chocolate brown or greyish-brown to dark brown or blackish-brown basidiomata, without violet tinge, chocolate brown to dark brown hymenophore, tubes not separable from the pileus context, white to off-white basal mycelium which turns reddish-white to pale red when bruised, amygdaliform to ovoid with subacute apex in side view to ovoid basidiospores and dark brown spore deposit.

#### Description.

***Basidiomata*** stipitate-pileate with poroid hymenophore, small to medium-sized, dull, brown to greyish-brown to dark brown or blackish-brown. ***Pileus*** convex when young becoming plano-convex to slightly depressed with age, with deflexed to inflexed margin; ***surface*** even to subrugulose, minutely tomentose or slightly cracked at the centre; ***context*** soft, yellowish to greyish off-white then slightly greyish-orange to dull orange to greyish-brown when exposed to the air, patchy or marmorated with greyish-brown to dark brown, sometimes with scattered small dark brown to brownish-black encrustations, not or inconsistently reddening when cut. ***Hymenophore*** tubulate, adnate, subventricose to ventricose, slightly depressed around the stipe; ***tubes*** brown to greyish-brown to dark brown, not separable from the pileus context; ***pores*** regularly arranged, mostly roundish at first becoming slightly angular with age, sometimes irregular, elongated around the stipe, dark brown to greyish-brown at first, becoming brown to chocolate brown with age. ***Stipe*** central, terete to sometimes slightly compressed, cylindrical to sometimes slightly wider at the base; surface even, minutely tomentose, dull, dark brown to greyish-brown, basal mycelium white to off-white becoming reddish-white to pale red when touched; ***context*** solid, yellowish to orange white to yellowish-grey to pale orange to dull orange to reddish-grey, marmorated or virgated with brownish-grey to greyish-brown to dark brown, sometimes scattered with small reddish-brown to brownish-black fine encrustations, unchanged or inconsistently reddening when cut. ***Spore print*** dark brown.

***Basidiospores*** amygdaliform to ovoid or ovoid with subacute apex in side view, thin-walled, smooth, slightly reddish to brownish hyaline in water, slightly yellowish to greenish hyaline in KOH or NH_4_OH, inamyloid. ***Basidia*** 4-spored, clavate to narrowly clavate without basal clamp connection. ***Cheilocystidia*** fusiform or cylindrical with obtuse apex, sometimes bent or sinuate, thin-walled, often scattered with small brownish-yellow to yellowish-brown crystals on the walls in KOH or NH_4_OH. ***Pleurocystidia*** narrowly fusiform with obtuse apex or cylindrical to narrowly subclavate, sometimes bent or sinuate, thin-walled, densely covered with small reddish-brown to brownish dark encrustations on the walls when observed in H_2_O, which are discoloured then dissolved in KOH or NH_4_OH. ***Pileipellis*** a trichoderm becoming tangled trichoderm to tomentum, composed of thin-walled hyphae; terminal cells mostly slightly sinuate cylindrical to irregular with rounded apex or clavate to elongated clavate. ***Stipitipellis*** a trichoderm to tangled trichoderm or disrupted hymeniderm, composed of loosely to moderately interwoven cylindrical hyphae anastomosing at places. ***Clamp connections*** not seen in any tissue.

#### Typus generis.

*Cacaoporus tenebrosus*

#### Distribution.

Currently known from Thailand.

#### Notes.

*Sutorius* most closely resembles the new genus. In the field, *Cacaoporus* is easily distinguished from the *Sutorius* by the following combination of characters: chocolate brown to dark brown to blackish-brown basidiomata, which are darker than in *Sutorius* and never purplish-brown like in *Sutorius* species; chocolate brown to dark brown hymenophore, which is much darker than in *Sutorius* and never reddish- to purplish-brown like in *Sutorius*; tubes that are not separable from the pileus context but can be separated in *Sutorius*; off-white basal mycelium that more or less turns red when bruised, which is never the case in *Sutorius*.

### Cacaoporus pallidicarneus

Taxon classificationFungiBoletalesBoletaceae

Vadthanarat, Raspé & Lumyong sp. nov.

MB829657

**Figure 2.**
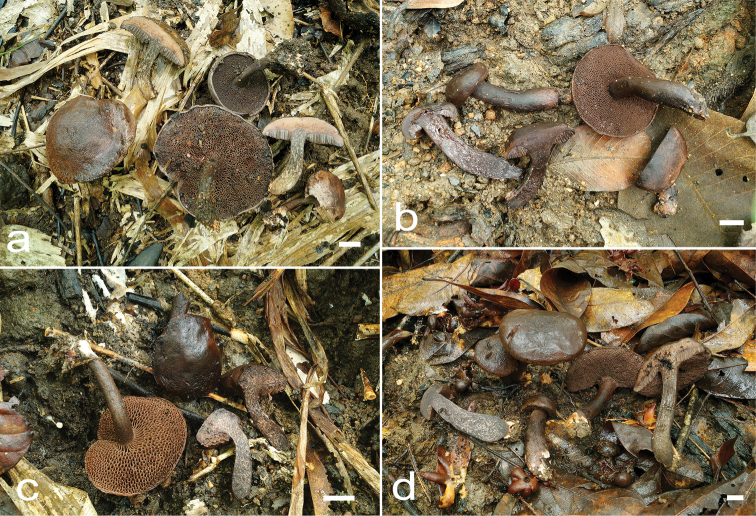
Habit of *Cacaoporus* species. **a***C.pallidicarneus* (SV0221) **b–d***C.tenebrosus* (b - SV0223, c - SV0224, d - SV0422). Scale bars: 1 cm (**a–d**).

**Figure 3.**
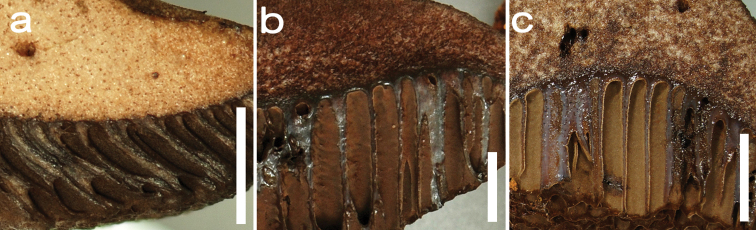
Close-ups of hymenium/pileus context transition zone in *Cacaoporus* species, illustrating the non-separability of both tissues **a***C.pallidicarneus* (OR0681) **b***C.tenebrosus* (OR0654) **c***C.tenebrosus* (SV0452). The transition between both tissues is particularly unmarked in *C.pallidicarneus* (**a**) Scale bars: 3 mm (**a**); 5 mm (**b–c**).

**Figure 4.**
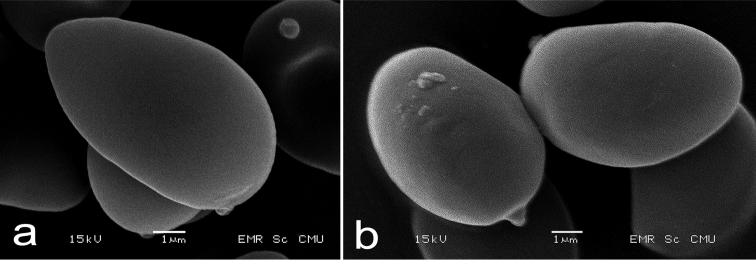
Scanning electron micrographs of *Cacaoporus* basidiospores **a***C.pallidicarneus* (SV0221) **b***C.tenebrosus* (SV0223). Scale bars: 1 µm (**a–b**).

**Figure 5.**
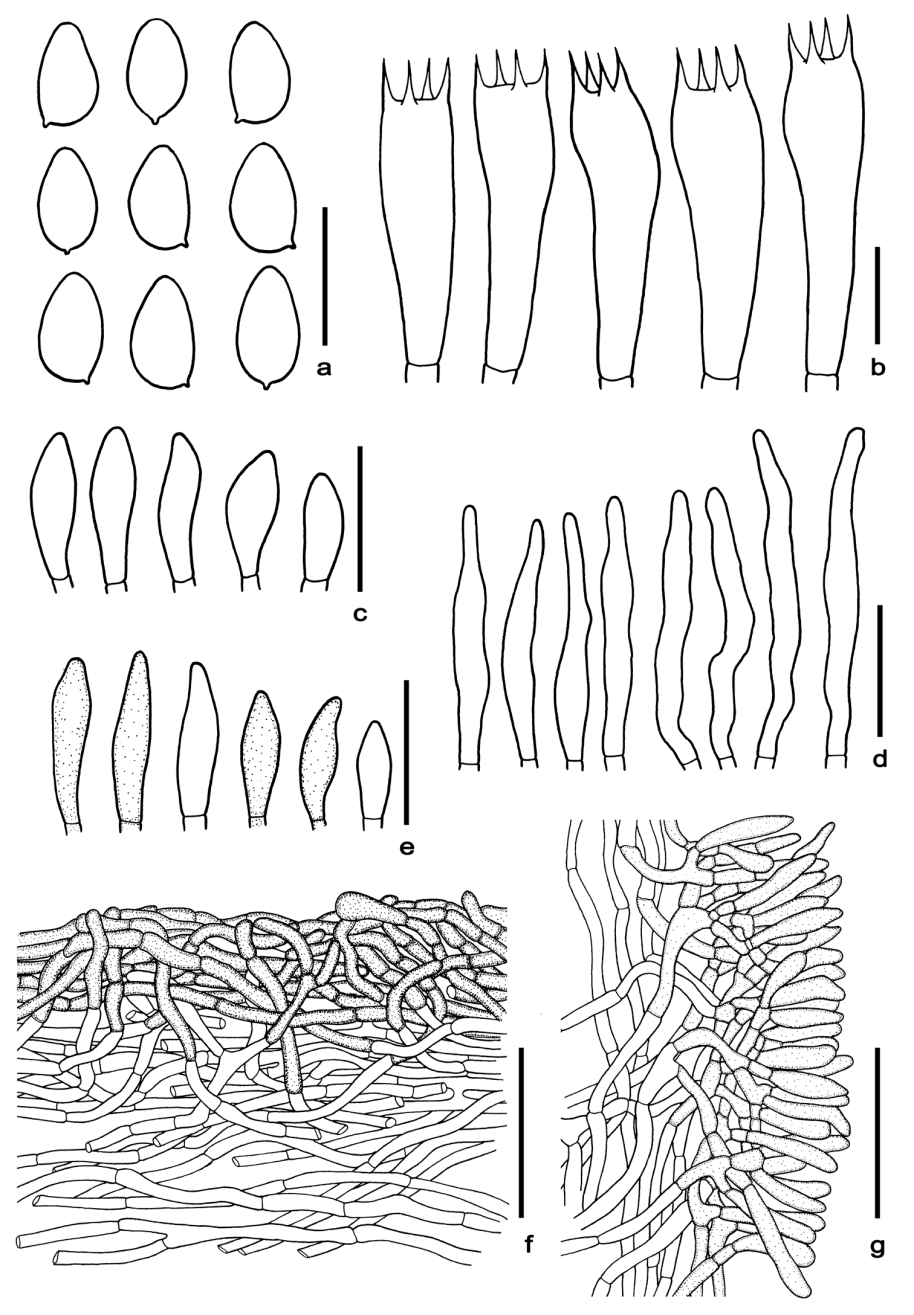
Microscopic features of *Cacaoporuspallidicarneus***a** basidiospores **b** basidia **c** cheilocystidia **d** pleurocystidia **e** caulocystidia **f** pileipellis **g** stipitipellis. Scale bars: 10 µm (**a–b**); 25 µm (**c–e**); 50 µm (**f–g**). All drawings were made from the type (SV0221).

Figs 2a , 3a , 4a and 5

#### Etymology.

Refers to the context, which is paler than in the other species, especially at the stipe base and in the pileus.

#### Type.

THAILAND, Chiang Mai Province, Mae On District, 18°52'37"N, 99°18'23"E, elev. 860 m, 15 August 2015, *Santhiti Vadthanarat*, SV0221 (CMUB!, isotype BR!).

#### Diagnosis.

*Cacaoporuspallidicarneus* is characterised by having a paler context than the other species and basidiospores that are amygdaliform or elongated amygdaliform to ovoid in side view, sometimes with subacute apex, shorter basidia and fusiform to narrowly bent fusiform to narrowly fusiform hymenophoral cystidia.

#### Description.

***Basidiomata*** small to medium-sized. ***Pileus*** (1.6)2.4–5.5 cm in diameter, convex when young becoming plano-convex with age; margin deflexed to inflexed, slightly exceeding (1–2 mm), surface even to subrugulose, minutely tomentose, dull, at first brown to greyish-brown to blackish-brown (8F3–4) sometimes paler (8C2) at places, becoming paler to greyish-brown (8E3–5) with age; ***context*** 4–9 mm thick half-way to the margin, soft, yellowish to greyish off-white then slightly pale orange to greyish-orange (6A3 to 6B3) when exposed to the air, with patchy or marmorated with greyish-brown (8E3) especially when young, scattered with reddish-brown to brownish-black of fine encrustations at places, slightly reddening when cut. ***Stipe*** central, terete or sometimes slightly compressed, cylindrical with slightly wider base, (2.0)2.8–3.7 × 0.4–0.7 cm, surface even, minutely tomentose, dull, greyish-brown to dark brown (8 E/F 3–4 to 8F2), basal mycelium white to off-white becoming pale red (7A3) when bruised; ***context*** solid, yellowish to greyish off-white then orange white to pale orange (5A2–3) when exposed to the air, virgate to marmorate with brownish-grey (8F2), less so at the stipe base, at places scattered with brownish-black fine encrustations, unchanged to slowly slightly reddening when cut. ***Hymenophore*** tubulate, adnate, subventricose, slightly depressed around the stipe. ***Tubes*** (2)4–6 mm long half-way to the margin, brown to greyish-brown (8F3), not separable from the pileus context. ***Pores*** 0.4–1.5 mm wide at mid-radius, regularly arranged, mostly roundish to elliptical at first, becoming slightly angular with age, slightly elongated around the stipe, colour distribution even, dark brown to chocolate brown (9F4 to 10F3) at first, becoming chocolate brown to brown (10F4 to 7–8F4–5) with age. ***Odour*** rubbery. ***Taste*** slightly bitter at first, then mild. ***Spore print*** dark brown (8F4/5) in mass.

***Macrochemical reactions.***KOH, orange brown on cap, yellowish-black on stipe, yellowish-black on the pileus context and stipe context, brownish-black on hymenium; NH_4_OH, yellowish-brown on cap, yellowish-orange on stipe, orangey yellow to yellowish-orange on the pileus context, stipe context and hymenium.

***Basidiospores*** [437/7/5] (6.5–)6.7–7.7–8.6(–11.5) × (3.8–)4–4.6–5.1(–5.5) µm *Q* = (1.4–)1.48–1.68–1.9(–2.44). From the type (3 basidiomata, *N* = 177) (6.8–)7–7.8–8.5(–9.1) × (4–)4.2–4.6–5(–5) µm, *Q* = (1.49–)1.5–1.69–1.9(–2.21), amygdaliform or elongated amygdaliform sometimes to ovoid with subacute apex in side view, ovoid in front view, thin-walled, smooth, slightly reddish to brownish hyaline in water, slightly yellowish to greenish hyaline in KOH or NH_4_OH, inamyloid. ***Basidia*** 4-spored, (25.3–)25.4–29.7–33.8(–33.8) × (7.3–)7.3–8.4–9.8(–10) µm, clavate without basal clamp connection, slightly yellowish to brownish hyaline in KOH or NH_4_OH; sterigmata up to 5 µm long. ***Cheilocystidia*** (16–)16.3–23.4–32.8(–34) × (5.5–)5.8–7.3–9(–9) µm, frequent, fusiform, thin-walled, yellowish to brownish hyaline to brown in KOH or NH_4_OH. ***Pleurocystidia*** (44–)44.2–54.7–67.6(–68) × (5–)5–6–7(–7) µm, frequent, usually narrowly bent fusiform to narrowly fusiform with obtuse apex, thin-walled, yellowish to brownish hyaline in KOH or NH_4_OH. ***Hymenophoral trama*** subdivergent to divergent, 62–175 µm wide, with 25–100 µm wide, regular to subregular mediostratum, composed of cylindrical, 4–7(11) µm wide hyphae, yellowish to brownish hyaline in KOH or NH_4_OH. ***Pileipellis*** a trichoderm to tangled trichoderm at first, becoming a tomentum to tangled trichoderm with age, 65–110 µm thick, composed of firmly to moderately interwoven thin-walled hyphae; terminal cells 12–55 × 4–6 µm, slightly bent cylindrical with rounded apex, at places clavate to sub-clavate to elongated clavate, 16–34 × 8–10 µm, slightly dark to reddish to brownish dark in water, yellowish to brownish hyaline to yellowish-brown to slightly dark at places in KOH or NH_4_OH. ***Pileus context*** made of moderately interwoven, thin-walled, hyaline hyphae, 6–12 µm wide. ***Stipitipellis*** a disrupted hymeniderm, 55–95 µm thick, composed clavate cells, 11–37 × 5–8 µm, yellowish-brown to slightly dark in KOH or NH_4_OH mixed with caulocystidia. ***Caulocystidia*** (17–)17–23.6–31(–31) × (5–)5–6.3–7(–7) µm, frequent, thin-walled, mostly yellowish-brown to slightly dark at places in KOH or NH_4_OH. ***Stipe context*** composed of parallel, 3–7 µm wide hyphae, brownish hyaline to yellowish pale brown in KOH or NH_4_OH. ***Clamp connections*** not seen in any tissue.

#### Habitat and Distribution.

solitary to gregarious up to 4 basidiomata, on soil in hill evergreen forest dominated by Fagaceae trees, with a few *Dipterocarpus* spp. and *Shorea* spp. or in Dipterocarp forest dominated by *Dipterocarpus* spp. and *Shorea* spp. with a few *Lithocarpus* sp., *Castanopsis* sp. and *Quercus* sp. Currently known only from Chiang Mai Province, Northern Thailand.

#### Additional specimens examined.

THAILAND, Chiang Mai Province, Mae Taeng District, 23 km marker (Ban Tapa), 19°08'50"N, 98°46'50"E, elev. 930 m, 2 August 2013, *Olivier Raspé* & *Anan Thawthong*, OR0681; Ban Mae Sae, 19°14'70"N, 98°38'70"E, elev. 960 m, 3 August 2013, *Olivier Raspé* & *Anan Thawthong*, OR0683; Muang District, Doi Suthep-Pui National Park, 18°48'37"N, 98°53'33"E, elev. 1460 m, 14 July 2016, *Olivier Raspé*, OR1306; Mae On District, 18°52'35"N, 99°18'16"E, elev. 860 m, 6 June 2018, *Santhiti Vadthanarat*, SV0451.

#### Remarks.

We observed some small yellowish to reddish to brownish dark particles or crystals covering the cell walls in pileipellis, stipitipellis and on the hymenium, especially the cystidia and basidia when observed in water. The small particles or crystals were mostly dissolved in KOH.

*Cacaoporuspallidicarneus* differs from *C.tenebrosus* by its basidiomata context colour which is paler, especially at the stipe base. A combination of the following characters are also distinctive: spore shape which is amygdaliform or elongated amygdaliform or sometimes ovoid with subacute apex in side view and ovoid in front view, while *C.tenebrosus* has ovoid spores, shorter basidia and differently shaped hymenophoral cystidia (see note under *C.tenebrosus*). *Cacaoporuspallidicarneus* has a stipitipellis which is a disrupted hymeniderm composed of caulocystidia and clavate cells, while the other species has a loose trichoderm or tangled trichoderm. Interestingly, one collection (SV0402) had a slightly paler context than *C.tenebrosus* but not as pale as *C.pallidicarneus*. The phylogenetic analyses indicated that this collection might be a species different from *C.pallidicarneus* and *C.tenebrosus*. However, the specimen was immature and, therefore, more collections are needed before the species can be formally recognised.

### Cacaoporus tenebrosus

Taxon classificationFungiBoletalesBoletaceae

Vadthanarat, Raspé & Lumyong sp. nov.

MB829656

**Figure 6.**
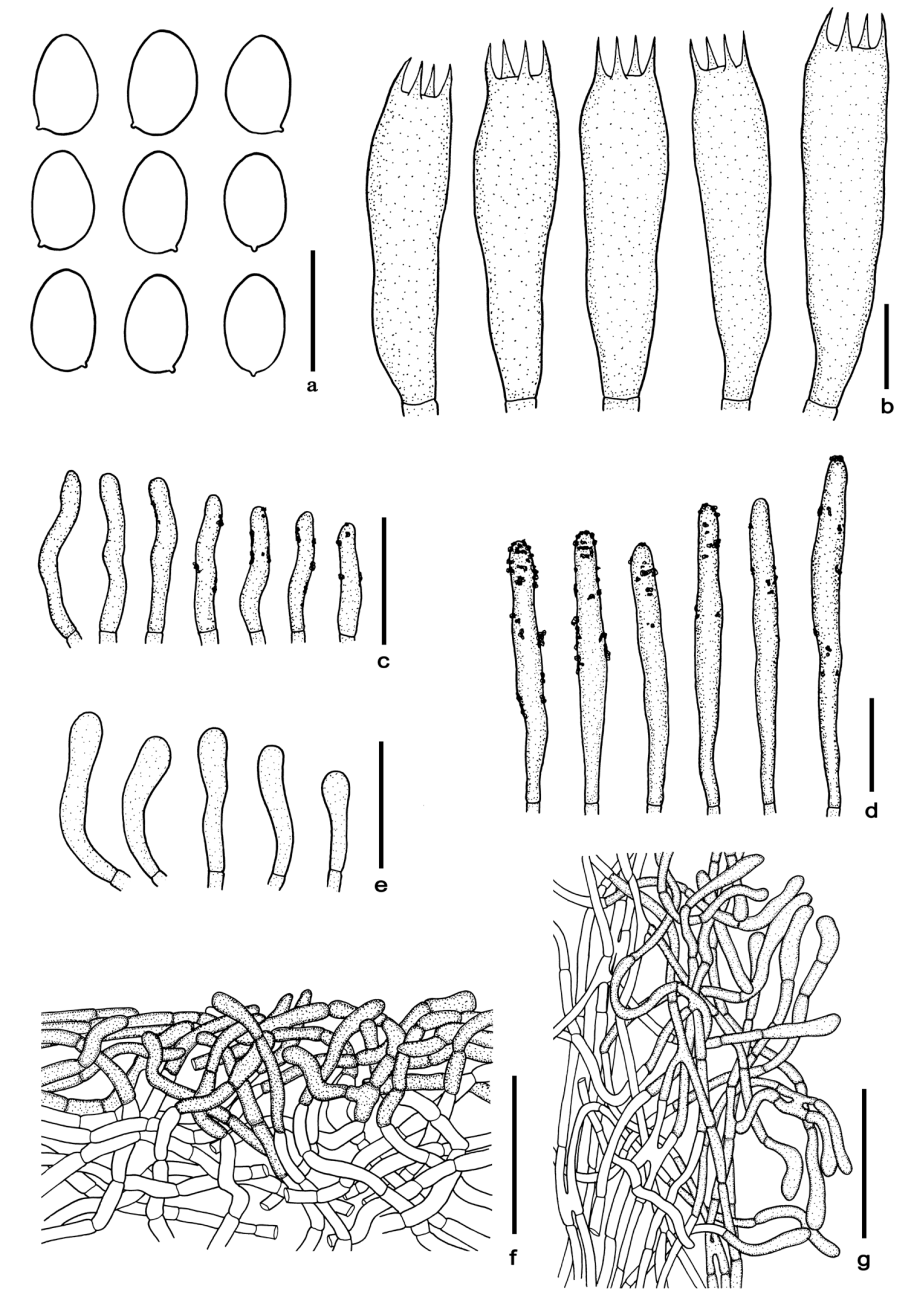
Microscopic features of *Cacaoporustenebrosus***a** basidiospores **b** basidia **c** cheilocystidia **d** pleurocystidia **e** caulocystidia **f** pileipellis **g** stipitipellis. Scale bars: 10 µm (**a–b**); 25 µm (**c–e**); 50 µm (**f–g**). All drawings were made from the type (SV0223).

Figs 2b–d , 3b–c , 4b and 6

#### Etymology.

Refers to the overall darkness of basidiomata, including the context.

#### Type.

THAILAND, Chiang Mai Province, Mae On District, 18°52'37"N, 99°18'32"E, elev. 940 m, 15 August 2015, *Santhiti Vadthanarat*, SV0223 (holotype CMUB!, isotype BR!).

#### Diagnosis.

*Cacaoporustenebrosus* is characterised by having a darker context than the other species, longer basidia and cylindrical to narrowly subclavate hymenophoral cystidia.

#### Description.

***Basidiomata*** medium-sized. ***Pileus*** (2.3)3.1–5(9) cm in diameter, convex when young becoming plano-convex to slightly depressed with age; margin inflexed to deflexed, slightly exceeding (1–2 mm); surface even to subrugulose, minutely tomentose, slightly cracked at the centre, dull, greyish-brown (10F3) to dark brown to blackish-brown (8F4–5) to the margin; ***context*** 5–10 mm thick half-way to the margin, soft, marmorated, greyish-brown to dark brown (10F3–5) with greyish-brown (9B/D3), scattered with reddish-brown to brownish-black, fine encrustations at places, slightly reddening in paler spots when cut. ***Stipe*** central, terete, cylindrical to sometimes with slightly wider base, 4.3–7.0 × 0.7–1.4 cm, surface even, minutely tomentose, dull, dark brown to greyish-brown (9F4 to 10F3), basal mycelium white to off-white becoming reddish-white to pale red (7A3–4) when bruised; ***context*** solid, greyish-brown to dark brown (9–10F3–5) marmorated with reddish-grey (7/10B2), usually scattered with small reddish-brown to brownish-black fine encrustations, slightly reddening when cut. ***Hymenophore*** tubulate, adnate, subventricose to ventricose, slightly depressed around the stipe. ***Tubes*** (4)7–13 mm long half-way to the margin, brown to dark brown (8F3 to 9F4), not separable from the pileus context. ***Pores*** 0.8–2 mm wide at mid-radius, regularly arranged, mostly roundish at first, becoming slightly angular with age, sometime irregular, elongated around the stipe; colour distribution even, greyish-brown to dark brown (9F4) at first, becoming chocolate brown to brown (10F3 to 7–8F4–5) with age. ***Odour*** mild fungoid. ***Taste*** slightly bitter at first, then mild. ***Spore print*** dark brown (8/9F4) in mass.

***Macrochemical reactions.***KOH, yellowish then brown to black on cap, stipe, pileus context, stipe context and hymenium; NH_4_OH, yellowish then orange to brown on cap, stipe, pileus context, stipe context and hymenium.

***Basidiospores*** [290/8/6] (7.4–)7.7–8.4–9.2(–10) × (4.5–)5–5.3–5.7(–6.1) µm *Q* = (1.25–)1.44–1.57–1.77(–2). From the type (2 basidiomata, *N* = 134) (7.5–)7.7–8.2–9(–9.9) × (4.9–)5–5.4–5.7(–5.9) µm, *Q* = (1.41–)1.43–1.54–1.71(–1.9), ovoid, thin-walled, smooth, slightly reddish to brownish hyaline in water, slightly yellowish to greenish hyaline in KOH or NH_4_OH, inamyloid. ***Basidia*** 4-spored, (33.6–)34.3–38.8–45.8(–47) × (7.7–)7.8–9.5–10.8(–10.9) µm, clavate to narrowly clavate without basal clamp connection, yellowish to brownish hyaline to slightly dark in KOH or NH_4_OH; sterigmata up to 5 µm long. ***Cheilocystidia*** (22–)22.1–28.7–37(–37) × (3–)3.1–4.4–5(–5) µm, frequent, cylindrical with obtuse apex, sometimes bent or sinuate, thin-walled, yellowish-brown to dark brown in KOH or NH_4_OH, often scattered with small brownish-yellow to yellowish-brown crystals on the walls in KOH or NH_4_OH. ***Pleurocystidia*** (62–)62.5–81.5–99(–99) × (7–)7–8–9(–9) µm, frequent, cylindrical to narrowly subclavate, sometimes bent or sinuate, thin-walled, with yellowish-brown to slightly dark content in KOH or NH_4_OH, densely covered with small reddish-brown to brownish dark encrustations on the walls when observed in H_2_O, with some scattered small brownish-yellow to yellowish-brown crystals on the walls in KOH or NH_4_OH. ***Hymenophoral trama*** subdivergent to divergent, 80–170 µm wide, with 60–80 µm wide of subregular mediostratum, composed of cylindrical, 4–8(11) µm wide hyphae, slightly yellowish to brownish hyaline in KOH or NH_4_OH. ***Pileipellis*** a tangled trichoderm to tomentum at places, 70–110 µm thick, composed of moderately interwoven thin-walled hyphae; terminal cells 12–48 × 4–7 µm mostly slightly sinuate, cylindrical to irregular with rounded apex, at places clavate to elongated clavate terminal cells 18–33 × 7–9 µm, slightly dark to reddish to brownish dark in water, yellowish-brown to slightly dark in KOH or NH_4_OH, scattered with small brownish-yellow to yellowish-brown crystals on the walls in KOH or NH_4_OH. ***Pileus context*** made of moderately interwoven, thin-walled, hyaline hyphae, 7–12 µm wide. ***Stipitipellis*** a trichoderm to tangled trichoderm, 70–120 µm thick, composed of loosely to moderately interwoven cylindrical hyphae anastomosing at places, brownish dark to dark in KOH or NH_4_OH. ***Caulocystidia*** (17–)17.6–29.4–46.3(–47) × (4–)4.1–5.5–6.9(–7) µm, clavate to cylindrical with obtuse apex, thin-walled, yellowish to brownish dark in KOH or NH_4_OH. ***Stipe context*** composed of parallel, 4–6(12) µm wide hyphae, brownish hyaline to yellowish pale brown in KOH or NH_4_OH. ***Clamp connections*** not seen in any tissue.

#### Habitat and distribution.

Gregarious (up to 9 basidiomata) to fasciculate or solitary, on soil in hill evergreen forest dominated by Fagaceae trees, with a few *Dipterocarpus* spp. and *Shorea* spp. or in Dipterocarp forest dominated by *Dipterocarpus* spp., *Shorea* spp. with a few *Lithocarpus* sp., *Castanopsis* sp. and *Quercus* sp. Currently known only from Chiang Mai Province, Northern Thailand.

#### Additional specimens examined.

THAILAND, Chiang Mai Province, Mae Taeng District, 19°07'15"N, 98°43'55"E, elev. 910 m, 29 July 2013, *Olivier Raspé* & *Benjarong Thongbai*, OR0654; ibid. 19°7'29"N, 98°40'59"E, elev. 1010 m, 24 May 2018, *Santhiti Vadthanarat*, SV0422; Mae On District, 18°52'37"N, 99°18'19"E, elev. 850 m, 15 August 2015, *Santhiti Vadthanarat*, SV0224; ibid., 18°52'35"N, 99°18'16"E, elev. 860 m, 15 July 2017, *Olivier Raspé* , OR1435; ibid., 6 June 2018, *Santhiti Vadthanarat*, SV0452.

#### Remarks.

There were many small yellowish to reddish to dark brownish particles or crystals on the walls of pileipellis, stipitipellis and hymenium cells, especially on the cystidia and basidia when observed in water. The small particles or crystals are somewhat dissolved and discoloured in KOH.

Microscopically, *Cacaoporustenebrosus* differs from *C.pallidicarneus* by having a darker context, longer basidia (33.6–47 µm vs. 25.3–33.8 µm, respectively), longer and larger hymenophoral cystidia, which also differ in shape (cylindrical to narrowly subclavate in *C.tenebrosus* but fusiform to narrowly fusiform in *C.pallidicarneus*). Phylogenetically, all *Cacaoporus* collections with a dark context formed a clade sister to *C.pallidicarneus* (BS = 85% and PP = 0.88), but some (SV0224 and SV0422) were genetically somewhat distant from the other collections. However, we could not find any difference in morphology. Consequently, we consider them as the same species (*C.tenebrosus*). Study of more collections is needed to confirm or infirm that they belong to the same species.

## Discussion

Morphologically, *Cacaoporus* is most similar to *Sutorius*, with which it shares the overall brown colour of basidiomata and encrustations in the flesh. However, the genus *Cacaoporus* has darker basidiomata, especially the hymenophore and pore surface and is more chocolate brown, not reddish-brown or purplish-brown like *Sutorius*, tubes that are not separable from the pileus context whereas they are easily separable in *Sutorius*, white to off-white basal mycelium which becomes reddish when bruised, whereas in *Sutorius*, the basal mycelium is more or less white and unchanging. *Cacaoporus* also produces dark brown spore deposits whereas in *Sutorius*, spore deposits are reddish-brown (Halling et al. 2012). Microscopically, the two genera differ in the shape of basidiospores, which is amygdaliform to ovoid or ovoid with subacute apex in side view in *Cacaoporus*, whereas *Sutorius* produces narrowly ellipsoid to ellipsoid or subfusoid to fusoid basidiospores. Phylogenetically, *Cacaoporus* and *Sutorius* are not closely related - the two genera belong in two different clades of the *Pulveroboletus* group.

Some species in *Porphyrellus* E.-J. Gilbert also have brown to dark brown to umber basidiomata similar to *Cacaoporus*. However, *Porphyrellus* differs from the new genus in having white to greyish-white hymenophore when young, becoming greyish-pink to blackish-pink with age, white to pallid context in pileus and stipe variably staining blue and/or reddish when cut and white basal mycelium that does not turn red when bruised (Wolfe 1979; Wu et al. 2016). Some species in *Strobilomyces* Berk also share some characters with *Cacaoporus*, including dark brown basidiomata, white to off-white basal mycelium that turns red when bruised and the context turning red when cut. However, *Strobilomyces* species clearly differ from *Cacaoporus*, especially in the pileus surface, which is coarsely fibrillose or shows conical to patch-like scales, in the hymenophore, which is whitish-cream or greyish-brown or vinaceous drab and stains reddish then blackish when bruised and also basidiospores, which are subglobose to obtusely ellipsoid with reticulation or longitudinally striate (Gelardi et al. 2012; Antonín et al. 2015; Wu et al. 2016). Moreover, *Porphyrellus* and *Strobilomyces* were phylogenetically inferred to belong in subfamily Boletoideae (Wu et al. 2014, 2016; Vadthanarat et al. 2018) distinct from *Cacaoporus*.

Phylogenetically, *Cacaoporus* was monophyletic and clustered in a well-supported clade with the genera *Cyanoboletus* and *Cupreoboletus* and one undescribed taxon, *Boletus* p.p. sp. (specimen voucher JD0693), belonging to the *Pulveroboletus* group of Wu et al. (2014, 2016). *Cyanoboletus* and *Cupreoboletus*, however, differ from *Cacaoporus* in important morphological characters. The former two genera have a yellow hymenophore and yellowish context and tissues instantly discolouring dark blue when injured, and olive-brown spore deposits (Gelardi et al. 2014, 2015; Wu et al. 2016). The undescribed taxon represented by the voucher specimen JD0693, which clustered within the same clade as *Cacaoporus*, *Cyanoboletus* and *Cupreoboletus*, is also morphologically very different from *Cacaoporus*, in having yellow tubes, reddish pores, pale yellow to off-white context and reddish-brown pileus and stipe.

Our survey on the diversity of Boletes in the north of Thailand has been conducted since 2012 and no *Cacaoporus* has been found in the forests at elevations lower than 850 m. *Cacaoporus* was found only between 850 m and 1460 m elevation. However, more collections are needed to confirm that the distribution of the genus is restricted to mid- to high-elevation forests and does not occur in the lower elevation, drier forests. Most collections were made from Fagaceae-dominated, evergreen hill forests. The dominant trees in these forests belong to the Fagaceae, including *Lithocarpus*, *Castanopsis* and *Quercus*, but some Dipterocarpaceae may also occur. At the lower end of its elevation range, however, *Cacaoporus* was also found in Dipterocarpaceae-dominated forests (in which Fagaceae, especially *Quercus* spp., also occurs). The Dipterocarpaceae trees include *Dipterocarpus*, namely *D.tuberculatus*, *D.obtusifolius* and *Shorea*, namely *S.obtusa* and *S.siamensis*. The listed trees have previously been reported as ectomycorrhizal hosts of Boletaceae (Moser et al. 2009; Desjardin et al. 2009, 2011; Hosen et al. 2013; Arora and Frank 2014; Halling et al. 2014; Wu et al. 2018) and presumably are also the hosts for *Cacaoporus*.

**Figure 7.**
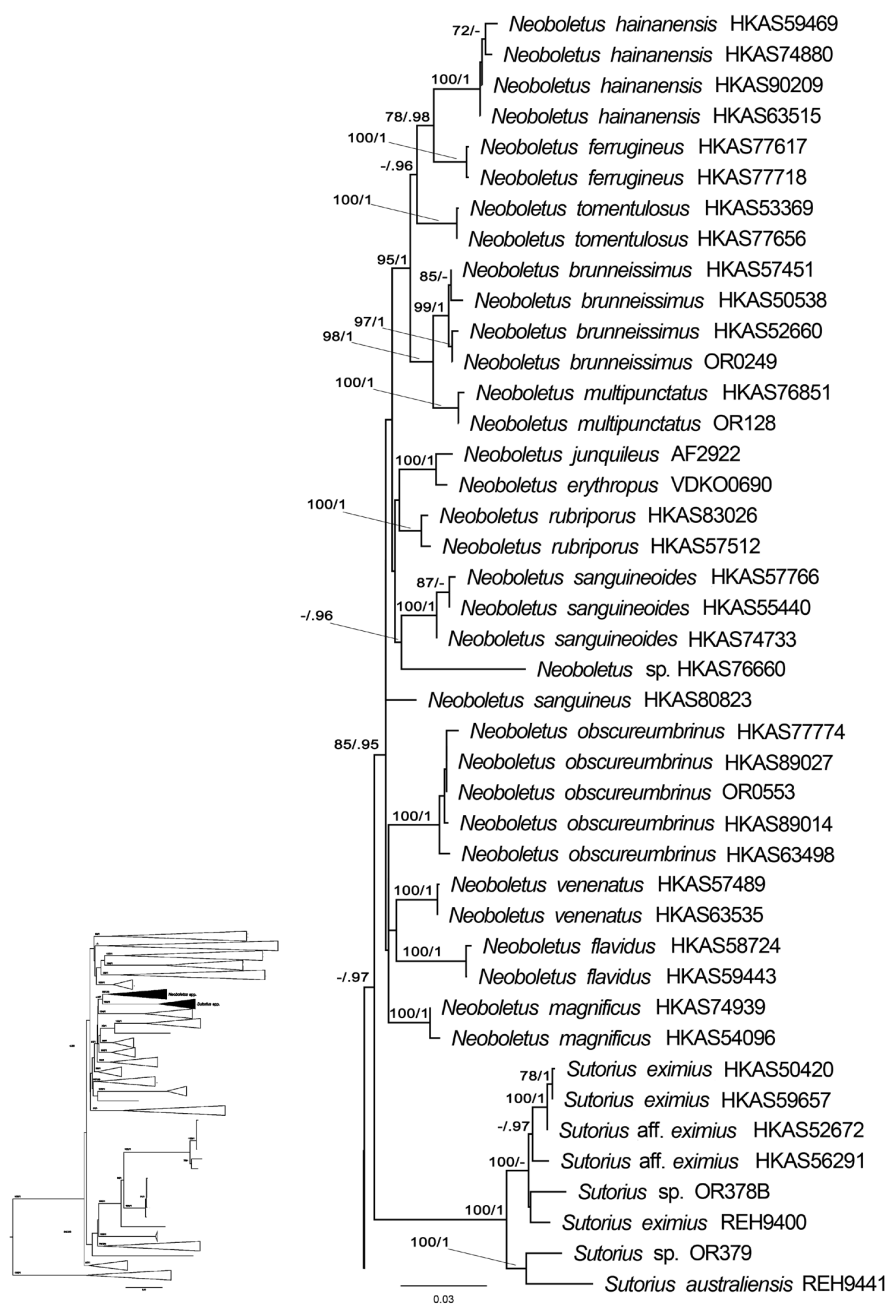
Sub-tree of the phylogram in Fig. 1, showing the well-supported *Sutorius* and *Neoboletus* clades and the unsupported sister relationship of *Neoboletusobscureumbrinus*.

Interestingly, our phylogeny indicated that the genera *Neoboletus* and *Sutorius* formed two different clades, both with high support (BS = 85% and PP = 0.95 for *Neoboletus*; BS = 100% and PP = 1 for *Sutorius*). Recently, Wu et al. (2016) synonymised *Neoboletus* with *Sutorius* because, in their phylogeny based on a four-gene dataset (28S+*tef*1+*rpb*1+*rpb*2), *Boletusobscureumbrinus*, a species morphologically more similar to *Neoboletus* than to *Sutorius*, seemed to cluster with *Sutorius* rather than with the *Neoboletus* species, although with neither ML nor BI support. Moreover, the *Neoboletus* clade was not supported either. Later, Chai et al. (2019) treated the two genera as different genetic lineages based on morphology and phylogeny (28S+ITS+*tef*1+*rpb*2), in which *B.obscureumbrinus* clustered with the other *Neoboletus* species in a well-supported clade. Our phylogenetic analyses, based on a different set of genes (*atp*6+ *tef*1+*rpb*2+*cox*3), confirm the separation of the two genera *Neoboletus* and *Sutorius*. The differences in gene trees obtained could be explained by a long-branch attraction artefact in datasets with different taxon and gene samplings and/or problems in the dataset (e.g. suboptimal alignment). *Neoboletusobscureumbrinus* is quite atypical amongst *Neoboletus* species and its phylogenetic affinities within this genus remain unclear (Fig. 7).

*Cacaoporus* is the second novel bolete genus described from Thailand, the first one being *Spongiforma* Desjardin, Manfr. Binder, Roekring & Flegel, described in 2009 (Desjardin et al.). However, fungal diversity in Thailand is high and still poorly known (Hyde et al. 2018), with a large number of species and possibly genera that remain to be described.

## Supplementary Material

XML Treatment for Cacaoporus

XML Treatment for Cacaoporus pallidicarneus

XML Treatment for Cacaoporus tenebrosus
